# A review of quartz crystal microbalance as a tool to monitor photocatalytic properties

**DOI:** 10.1039/d6ra06671b

**Published:** 2026-09-18

**Authors:** K. R. Jaliya Manuda, Dilushan R. Jayasundara

**Affiliations:** a Department of Physics, University of Colombo Colombo 03 Sri Lanka dilushanj@phys.cmb.ac.lk

## Abstract

Quartz crystal microbalance (QCM) is a versatile and sensitive technique for monitoring small interfacial mass changes through variations in the resonance frequency of a piezoelectric quartz crystal. Photocatalytic processes, particularly interfacial degradation and photoreduction, can therefore be monitored *in situ* and in real-time using QCM. This review summarizes QCM-based photocatalytic studies reported over the past two decades, covering both fundamental and application-oriented investigations involving a range of photocatalytic materials and organic or inorganic species. A structured literature search and selection strategy was used to identify and categorize reported QCM-based photocatalytic studies and to critically evaluate their experimental approaches and findings. Particular attention is given to quantitative comparison, experimental reproducibility, methodological limitations, and factors that can complicate the interpretation of QCM responses during photocatalysis. Emerging opportunities involving QCM-D, *operando* measurements, multimodal characterization, microfluidic platforms, and advanced acoustic resonators are also discussed.

## Abstract 2

Quartz crystal microbalance (QCM), a versatile technique to monitor and evaluate photocatalytic processes *in situ*, and in real-time.

## Graphical abstract

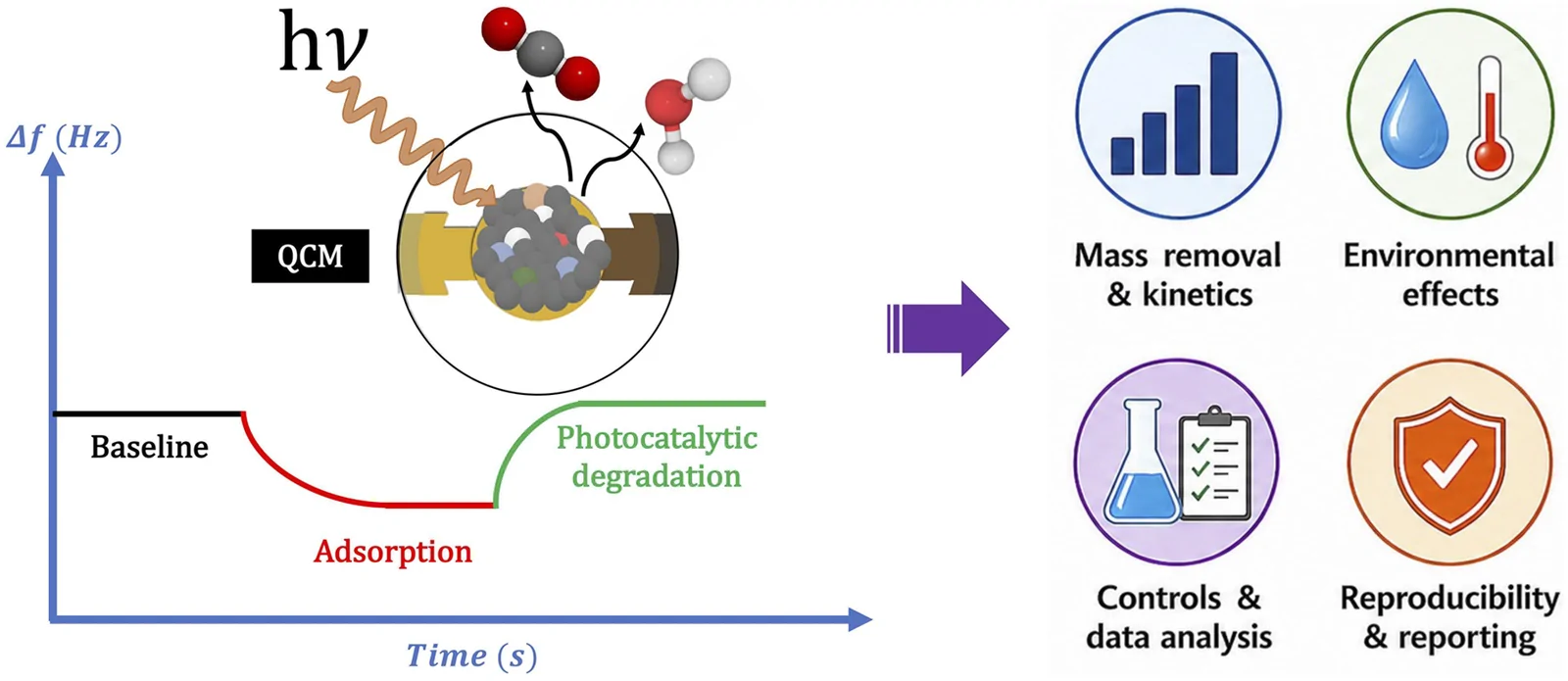

## 1. Introduction

Quartz crystal microbalance (QCM) is a highly sensitive, practical, and versatile analytical tool for monitoring small changes in interfacial mass through variations in the resonant frequency of a piezoelectric quartz crystal.^1^ Over the past few decades, QCM has emerged as a robust and widely recognized technique for a broad range of measurements, providing valuable insights into various properties of materials.

The history of QCM technology dates back to 1880 when Jacques and Pierre Curie formulated the theory of piezoelectricity.^2^ In 1959, Günter Sauerbrey investigated the relationship between mass adsorption on a quartz surface and the resulting frequency shift at the solid–gas interface.^3^ In the late 1980s, researchers discovered that by addressing the challenges posed by significant viscoelastic properties and moisture, the QCM could be effectively utilized in liquid environments, where these factors greatly influence its resonance behavior.^4^ As a result, QCM is now successfully operated in various liquid media, opening new opportunities to study the solid–liquid interface of materials.^5^

The piezoelectric effect in a thin quartz crystal, as utilized in QCM, involves two electrodes. This effect refers to generating an electrical potential within the material when subjected to mechanical stress.^2^ Conversely, the application of an electric field can induce mechanical deformation. The quartz crystal oscillates when an alternating electric field is applied to the electrodes. These oscillations generate a transverse acoustic wave, which propagates through the crystal and reflects off its surface.^6^ When the combined thickness of the crystal and the electrodes matches the acoustic wavelength, a standing wave is formed. This results in a resonance condition which is highly sensitive to changes in the mass loading and acoustic properties of the crystal-electrode system.^7^

The electrodes of the QCM are vacuum deposited with a thickness of about 100 nm,^8^ and made of gold (Au), silver (Ag), platinum (Pt), or titanium (Ti).^5,6^Fig. 1 shows a QCM with gold electrodes and the AT-cut of 35°15′ angle from the *Z*-axis of the quartz crystal. This is considered the best cutting angle to improve stability under ambient temperatures.^9^

**Fig. 1:**
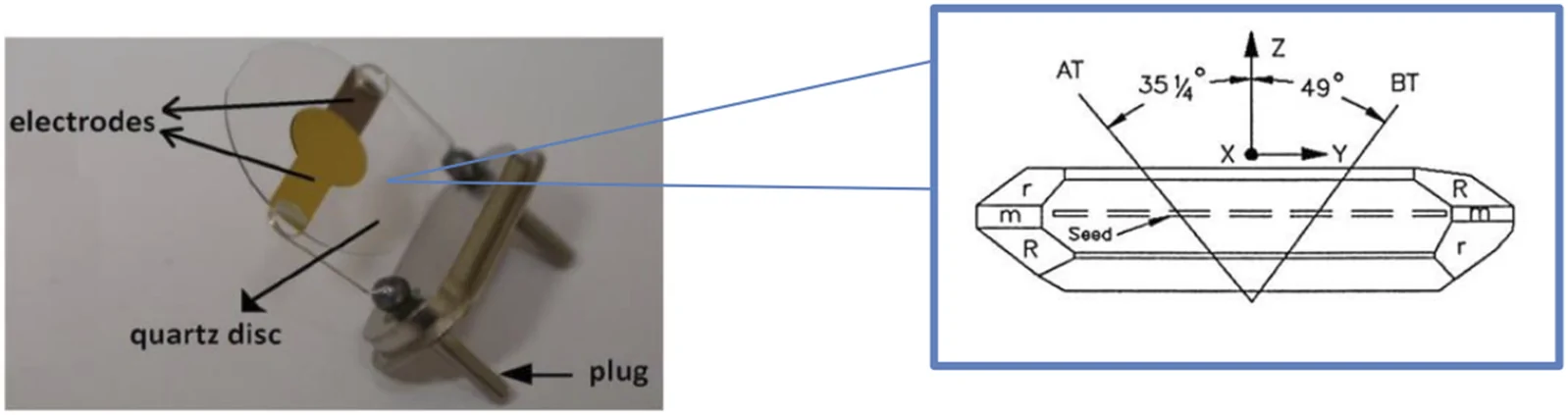
A typical AT-cut QCM with upper and lower faces consists of gold electrodes (Reprinted with permission from ref. 5 Copyright 2022 The Authors.).

The principle of the QCM technique is based on the inverse correlation between mass changes on the quartz crystal surface and the corresponding changes in its resonant frequency. This relationship is quantitatively described by the Sauerbrey equation:
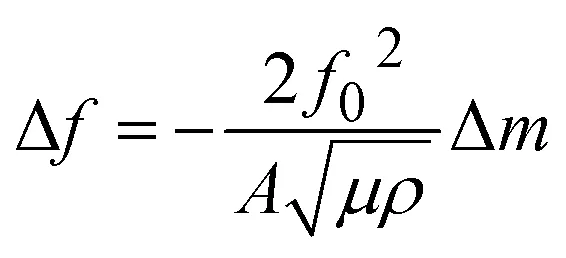
where Δf is the normalized frequency shift, Δm is the surface mass change, *f*_0_ is the frequency of the crystal's harmonic resonant mode, *A* is the piezoelectrically active area, *ρ* and *µ* are the density and shear modulus of quartz, respectively.^3,8^ The validity of the Sauerbrey equation is generally restricted to sufficiently thin, rigid, laterally homogeneous and acoustically well-coupled films for which energy dissipation is negligible.^9^ For soft, viscoelastic, porous, hydrated, or weakly coupled layers, the frequency response may contain significant contributions from changes in the mechanical and hydrodynamic properties of the film, and direct mass estimation using the Sauerbrey equation may therefore become unreliable. Moreover, the mass sensitivity function of a QCM is influenced by the material properties, geometry, thickness, and dimensions of the metal electrodes, as these factors affect the acoustic wave distribution and overall sensitivity to mass variations.^10,11^

Fig. 2 shows the top view and the cross-sectional view of a typical QCM sensor, followed by the expected theoretical variation of frequency change against time with increasing surface mass, according to Sauerbrey.^12^ Conversely, if the deposited mass on the surface is depleted over time, the frequency change must be increased in the positive direction.^8^ The plateauing effect may indicate that the QCM surface has attained an equilibrium, reached an experimental threshold, or other specific conclusion, which entirely depends on the nature of the experiment it is involved in.^13–15^

**Fig. 2:**
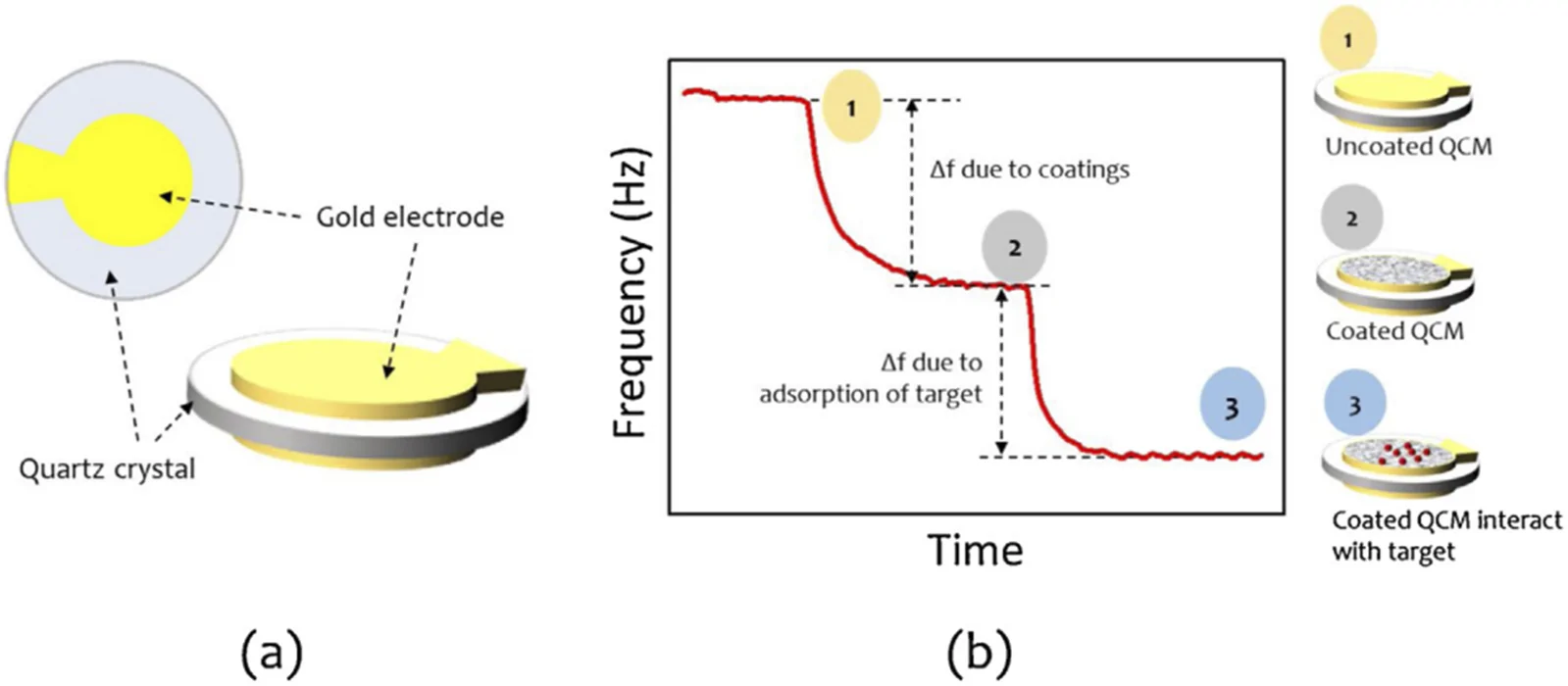
QCM sensor; (a) schematic top view and cross-sectional view (b) frequency changes that occur when coating and during sensing measurements (Reprinted with permission from ref. 12 Copyright 2021 Elsevier.).

For many years, the QCM functioned primarily as a general mass detector. The key challenge was to modify the surface chemistry to enhance its specificity, enabling the device to function as a selective and discriminating mass detector.^5^ However, since the beginning of the 2010 decade, a drastic increment in research based on QCM data is identified.^16^ This could be originated from the rapid advancement of information technology. QCM results are utilized in a vast variety of fundamental and application-based research studies, including but certainly not limited to, biosensor development,^1,6,9^ humidity and gas sensing applications,^14,17–19^ photocatalytic studies,^8,20–22^ protein immobilization studies,^23^ stress/strain applications,^24^ space system uncontrolled pollution studies,^25^ thermodynamic investigations,^26^ adsorption kinetic studies,^13,15^ and numerous medicinal applications.^6^

Despite being critically and continuously reviewed for chemical, biochemical, biological, and medicinal applications,^5,6,9,25,27–31^ the utilization of QCM in analyzing photocatalytic properties is not yet comprehensively reviewed. Nanomaterials such as graphene (GR), graphene oxide (GO), nano titanium dioxide (TiO_2_), nano zinc oxide (ZnO), hydroxyapatite (HA), and various nanocomposites^8,20–22,32–35^ have been studied using QCM for their photocatalytic properties.^36–41^ However, direct monitoring of these processes on coatings remains relatively unexplored. Limited studies have employed specialized experimental approaches to address this challenge; however, these methods often lack the sensitivity required to detect molecular-level changes.^42–44^ QCM is a particularly useful tool for investigating the photocatalytic properties of nanomaterials owing to its ability to directly monitor nanogram-level mass fluctuations, at both solid–air and solid–liquid interfaces.^14,15^ Furthermore, QCM can be operated under both *in situ* and *ex situ* conditions, enabling real-time continuous monitoring whenever needed.^8,45^ Moreover, nanocoatings can be readily deposited onto QCM substrates using spin-coating, electrophoretic deposition, dip-coating, electrospray deposition, and thin film sputtering.^27^ Thus, this review paper presents an overview of using the QCM method to study the photocatalytic properties of nanomaterials. We anticipate that this paper will provide valuable insights into this underexplored application of QCM.

## 2. Literature search and selection strategy

A literature search was conducted using Google Scholar to identify published studies investigating photocatalytic processes using the QCM technique. The primary search terms used were “QCM”, “photocatalytic”, “photocatalyst”, and “quartz crystal microbalance”. The retrieved literature was subsequently screened based on its relevance to the direct application of QCM for monitoring or evaluating photocatalytic processes.

Studies were considered relevant when QCM or QCM-D (QCM with dissipation monitoring) was used to monitor a photocatalytic process through changes in interfacial mass, resonance frequency, dissipation, or related acoustic responses. Particular attention was given to studies in which photocatalytic degradation or photoreduction was monitored *in situ* or in real-time. Studies involving QCM solely for conventional sensing, adsorption, surface characterization, or non-photocatalytic applications were not included in the principal photocatalytic assessment. The identified studies were categorized according to the photocatalytic interface, namely solid-air and solid–liquid systems. For each study, information relating to the QCM configuration, photocatalyst, pollutant, coating/deposition method, irradiation conditions, monitoring mode, and reported photocatalytic response was extracted where available. Quantitative parameters such as mass change, pollutant loading, degradation rate, rate constant, irradiation intensity, photocatalyst thickness, and QCM mass sensitivity were recorded when explicitly reported by the original authors.

Since substantial differences exist among the reported QCM configurations, photocatalysts, pollutants, irradiation conditions and kinetic models, quantitative comparison was restricted to studies for which the relevant parameters were sufficiently compatible. The reported data are critically compared to identify experimentally supported trends, methodological limitations, and opportunities for improving the reliability and comparability of QCM-based photocatalytic measurements.

## 3. Use of QCM in measuring photocatalytic properties

Literature shows that the major attention of QCM-based photocatalytic measurements is focused on photocatalytic degradation of organic pollutants using nanomaterials.^8,17,20–22,32–35,46–52^ The general mechanism of photocatalysis has been extensively investigated through both theoretical and experimental approaches.^53–57^ When a photon with energy equal to or greater than the band gap energy (*E*_g_) of a semiconductor (*hν* ≥ *E*_g_) interacts with the material, it promotes an electron (e^−^) from the valence band (VB) to the conduction band (CB), simultaneously generating a hole (h^+^) in the VB. The excited CB electrons and VB holes may undergo various processes, including recombination, where the input energy dissipates as heat, trapping in metastable surface states, or interactions with electron donors and acceptors adsorbed on the semiconductor surface or within the surrounding electrical double layer.^58^ The general photochemical reactions that start the oxidative and reductive pathways are listed below.^59,60^H_2_O + h^+^(VB) → H^+^ + OH˙O_2_ + e^−^(CB) → O_2_^˙−^2H_2_O + 2O_2_^˙−^ → H_2_O_2_ + 2OH˙ + O_2_H_2_O_2_ + e^−^(CB) → OH˙ + OH^−^

The species such as highly reactive OH˙ and O_2_^˙−^ radicals, photogenerated electrons and holes, and several other reactive oxygen species, including H_2_O_2_, ^1^O_2_, and ˙HO_2_ that are produced during photocatalysis contribute to the degradation of organic molecules in the surrounding area.^61–65^ These active species are generally referred to as reactive oxygen species (ROS).^8^

Typical steps of a QCM photocatalytic assessment are depicted in Fig. 3. The unloaded QCM has a resonant frequency of *f*_0_. When the photocatalytic material is coated as a film, the frequency is decreased by Δ*f*_Photo_ and further decreased by Δ*f*_org_ when an organic pollutant is adsorbed onto the photocatalytic film. Subsequently, the QCM is continuously illuminated by a desired light source, where a positive frequency shift followed by a plateauing effect is expected. This shift is expected to be lower than or equal to Δ*f*_org_, as it corresponds to the photocatalytic degradation of adsorbed organic molecules to form water and CO_2_ as degradation products. In practical photocatalytic measurements, however, the observed frequency response may be influenced by additional processes, including adsorption or desorption of water and other interfacial species, temperature variations, changes in film viscoelasticity, and the formation or removal of reaction intermediates. These factors can complicate the direct interpretation of frequency changes as pollutant degradation and are discussed in the subsequent sections.

**Fig. 3:**
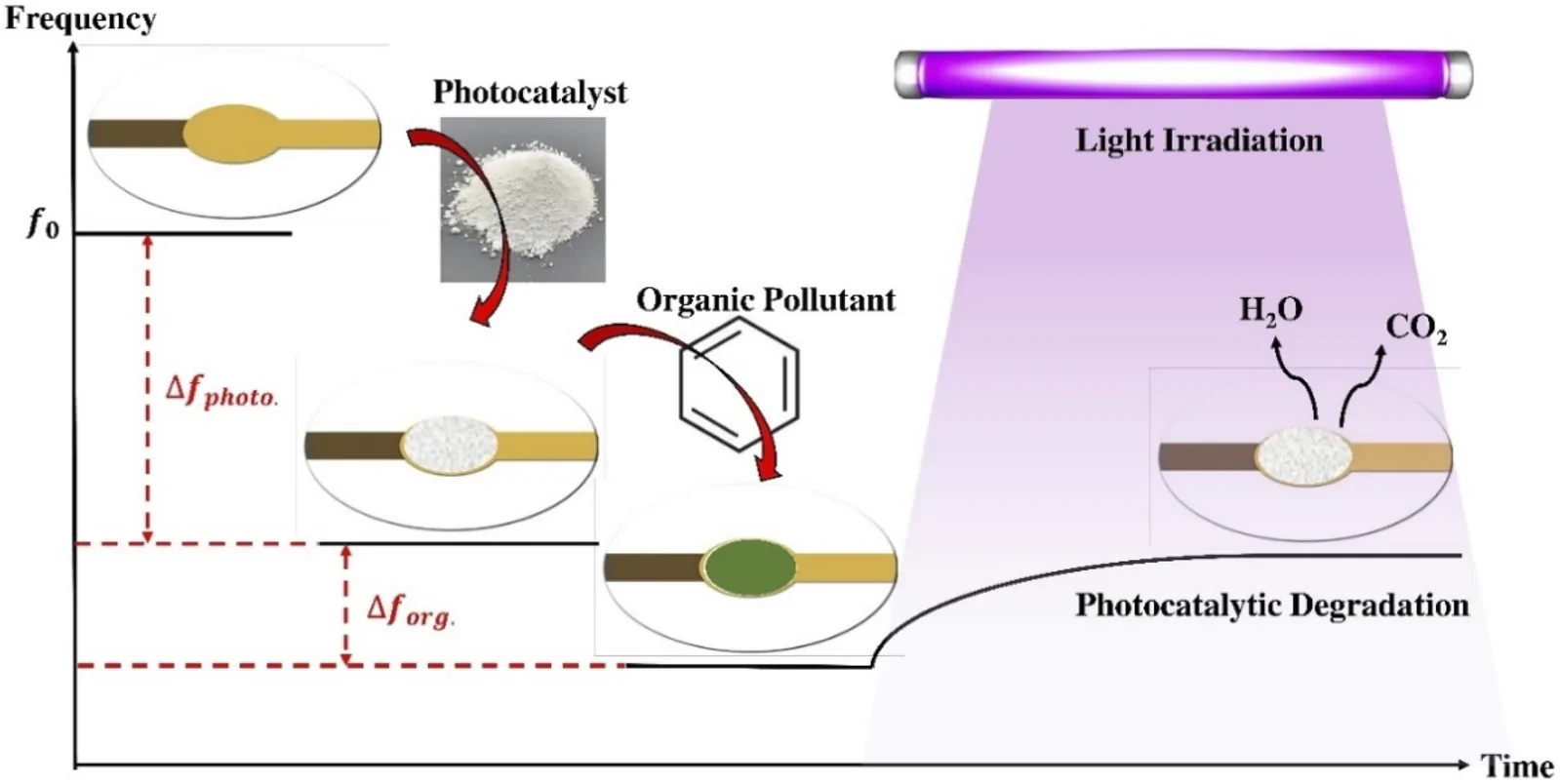
Idealized QCM frequency response during a typical photocatalytic experiment.

## 4. Photocatalytic assessments

There are two types of photocatalytic assessments carried out using the QCM method.

(a) Photocatalysis at the solid-air interface – QCM is operated under air mode.

(b) Photocatalysis at the solid–liquid interface – QCM is operated under liquid mode.

While each type has its advantages and disadvantages, both would yield quantitative data that can be analyzed to come to reliable conclusions.

### 4.1 Solid–air interface

In this configuration, the QCM is operated without immersion in a liquid phase, and the photocatalyst-coated surface is exposed to the surrounding gas or atmosphere throughout the measurement. This configuration can be employed when (a) the organic pollutant is in gaseous form or (b) a solid, liquid, or aqueous pollutant is deposited onto the photocatalyst-coated QCM by drop casting, spin-coating, or another suitable method. When the pollutant is introduced in the gas phase, QCM can simultaneously provide information on adsorption and subsequent photocatalytic degradation. Under ambient conditions adsorption and desorption of atmospheric water may also contribute to the QCM response and should therefore be considered.

#### 4.1.1 Gaseous pollutants

The first study of photocatalytic degradation of gaseous pollutants by employing the QCM method was reported in 2004 by Nakamura *et al.*^35^ They employed vaporized acetaldehyde and toluene under UV irradiation to evaluate the sensor properties of QCM. Nakamura *et al.* fabricated a 6 MHz Pt QCM with TiO_2_ using the spin-coating technique, which was then heat treated at 300 °C. The experiment was carried out in a chamber filled with nitrogen gas, into which the vaporized pollutants were injected. A black light (UV) with an irradiance of 1 mW cm^−2^ was used to illuminate the QCM. In their first experiment, they studied the adsorption of pure nitrogen and the nitrogen + acetaldehyde mixture in the absence and presence of UV irradiation. The results indicate that in the absence of UV, the QCM mass gain for the nitrogen + acetaldehyde mixture is higher than that for pure nitrogen, which is due to strong secondary interactions between acetaldehyde and the TiO_2_ surface. Subsequently, when the QCM is irradiated, a distinct mass loss was observed for the nitrogen + acetaldehyde mixture, which was assigned to the photocatalytic degradation of adsorbed acetaldehyde. Both nitrogen and the nitrogen + acetaldehyde mixture showed gradual mass gains even in the presence of UV, owing to the continuous adsorption of gas molecules. The results indicate that nitrogen adsorption was not significantly affected by UV irradiation, whereas acetaldehyde was removed from the surface under irradiation. This experiment was carried out *in situ* and in real-time, while the frequency readings were recorded at specific time intervals.

In 2009, Joo *et al.*^17^ employed the QCM method to evaluate the photocatalytic degradation of hexanethiol. They first directly grew ZnO nanorods with lengths of 0.9 µm (Z09) and 2.0 µm (Z20) on 10 MHz Au QCMs using a hydrothermal method. Subsequently, they monitored the continuous, *in situ* real-time adsorption of hexanethiol vapor on above prepared QCMs and a plane ZnO-sputtered QCM (Z0). Once the frequency variation was plateaued, adsorbed masses were calculated as 59.0 ng, 307.4 ng, and 625.7 ng for Z0, Z09, and Z20, respectively. This suggests that the adsorption increases with increasing surface area of the adsorbent.

Afterward, Joo *et al.* illuminated the above hexanethiol-adsorbed QCMs with a UV lamp, under the irradiance value of 4.5 mW cm^−2^. However, this was not monitored continuously in real-time. They paused the UV irradiation every 10 min, waited for the QCM response to settle, and then repeated until complete QCM-detectable removal of the deposited mass. The results of this study are shown in Fig. 4. All three QCMs display complete photocatalytic degradation of hexanethiol, while Z20 shows the best results as a self-cleaning thiol sensor. Z20 was subjected to an XPS analysis to confirm the complete removal of the gaseous pollutant.

**Fig. 4:**
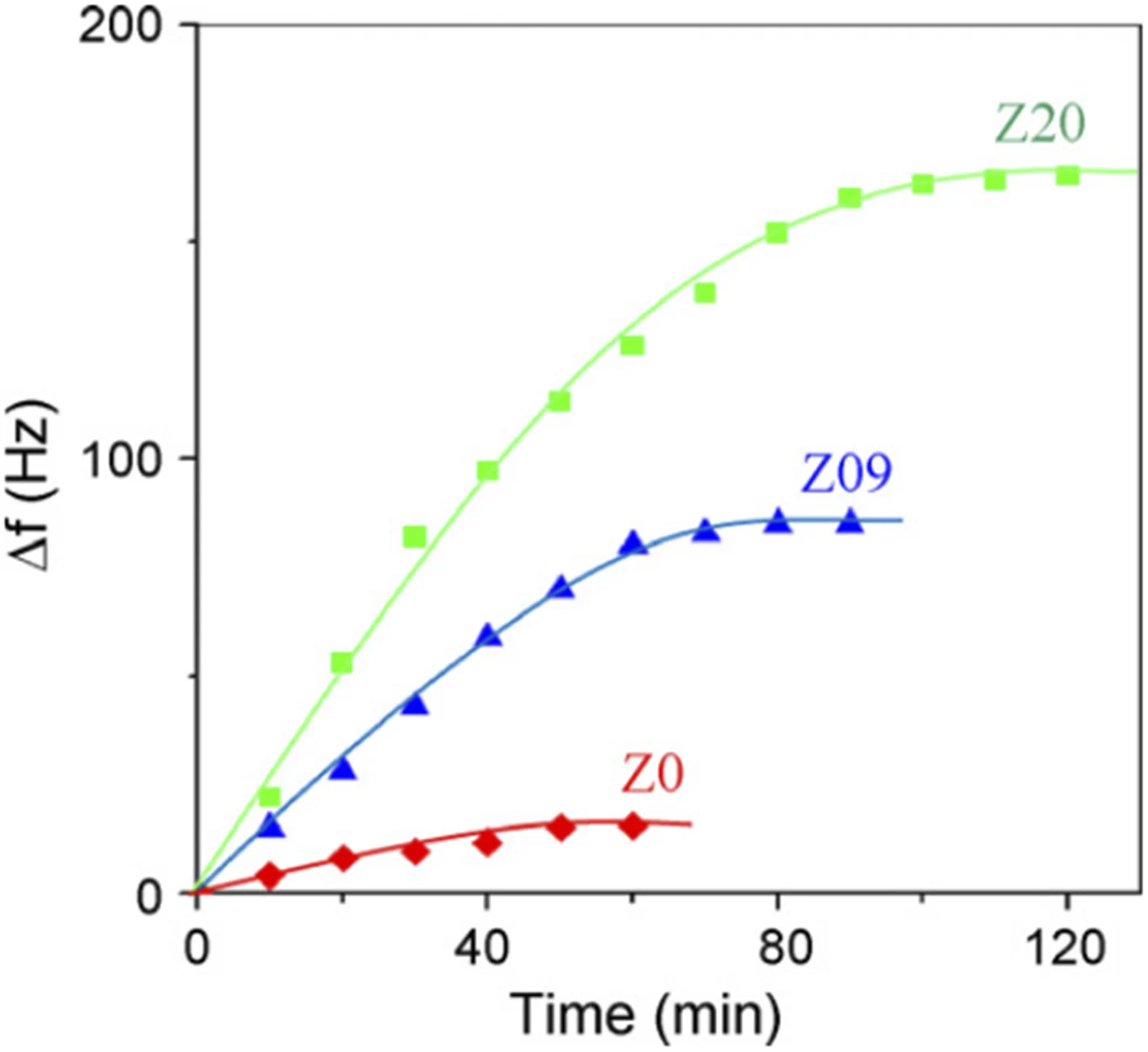
Variation of QCM frequency change against UV irradiation time in the photocatalytic degradation of hexanethiol (Reprinted with permission from ref. 17 Copyright 2009 Elsevier).

QCM method was further used to check the reusability and concentration–response correlation of Z20. This study is comprehensive with well-utilized QCM operations at the solid-air interface, except for the photocatalytic degradation measurements due to irradiation halts. Joo *et al.* explained the reason behind the halts after each 10 min of irradiation, as they observed a rise in temperature during irradiation.

In 2015, QCM technique was incorporated in a study reported by Qiang *et al.*^47^ with the objective of achieving a coating that could be switched between superhydrophilic and superhydrophobic characteristics, to be vitally associated with photocatalytic self-cleaning. Qiang *et al.* used a 6 MHz Au QCM, coated with a superhydrophobic TiO_2_/ZnO nanorod-composite-film, to measure the photocatalytic activity against acetone vapor. After surface wettability tests that proved superhydrophobicity of the coating, they subjected a bare QCM and a QCM coated with TiO_2_/ZnO nanorod-composite-film to the adsorption and photocatalytic experiments. Qiang *et al.* designed the QCM experiment in three steps, *i.e.* cleaning the QCMs with nitrogen, injecting acetone vapor into the vessels, and subsequent UV irradiation of the QCMs. The results of this study are shown in Fig. 5. The bare QCM adsorbed a considerable amount of acetone, however, the adsorbed amount was more than two-fold in the case of the nanomaterial due to higher surface area. The observed removal of adsorbed acetone under UV irradiation on the coated QCM, compared with the bare QCM, was attributed to the photocatalytic activity of the synthesized nanomaterial.

**Fig. 5:**
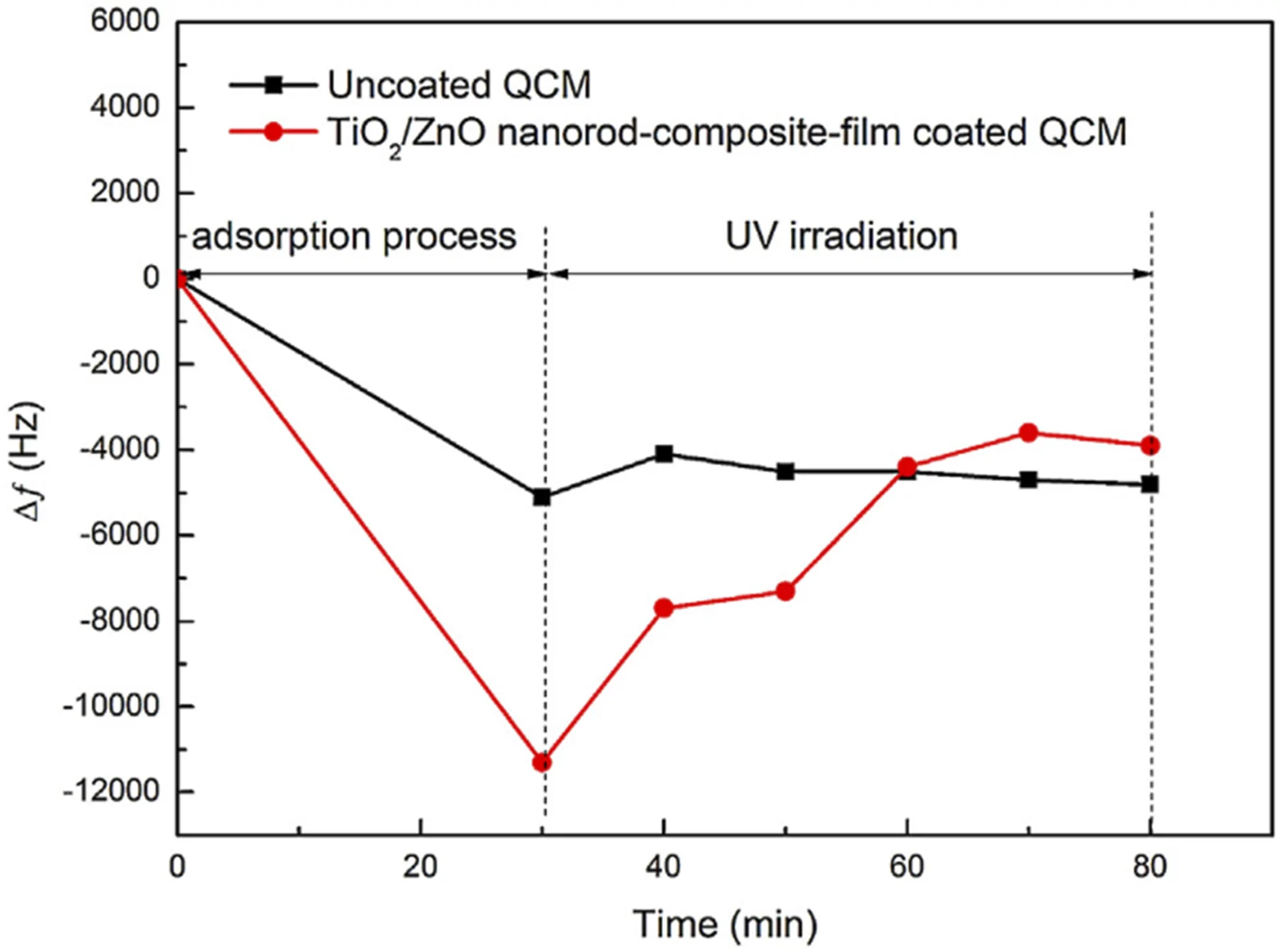
Adsorption and photocatalytic degradation of acetone on bare QCM and nanorod-composite-film coated QCM (Reprinted with permission from ref. 47 Copyright 2015 Elsevier).

The main objective was also fulfilled, as 2 h of UV (253.7 nm) irradiation transformed the surface from superhydrophobic (155° contact angle) to superhydrophilic (0° contact angle). This could be reversed back to superhydrophobicity by storing 7 days in the dark. Qiang *et al.* systematically demonstrated that surface hygroscopic properties can be tuned through photo irradiation, providing a comprehensive overview of this modulation mechanism.

Henry *et al.* published two QCM studies in 2020,^50^ and 2021,^66^ on photocatalytic degradation of paraffin oil vapor. Since the methodologies of these studies are similar, this review is focused on the first work, which discusses more details on the experimental setup. The second work is discussed later in brief.

In the first study,^50^ Henry *et al.* fabricated a TiO_2_ coating on a 6 MHz Cr/Au QCM, using the atomic layer deposition (ALD) technique. A custom-made experimental setup was employed to measure the adsorption and photocatalytic degradation of paraffin. This setup consisted of an as-coated QCM (QCM_1_) and a bare QCM (QCM_2_) mounted next to each other in a high vacuum chamber. Each QCM was connected to a separate frequency counter. The QCM experiment was performed in four stages. First, a vacuum of 1.6 × 10^−6^ mbar was applied (pumping stage). Next, paraffin oil was allowed to deposit on the two QCMs under vacuum (contamination stage). Subsequently, air venting followed by a short stabilization period was applied (venting stage). Then, the UV irradiation was started (illumination stage). The results of this continuously monitored, *in situ*, and real-time QCM study are depicted in Fig. 6.

**Fig. 6:**
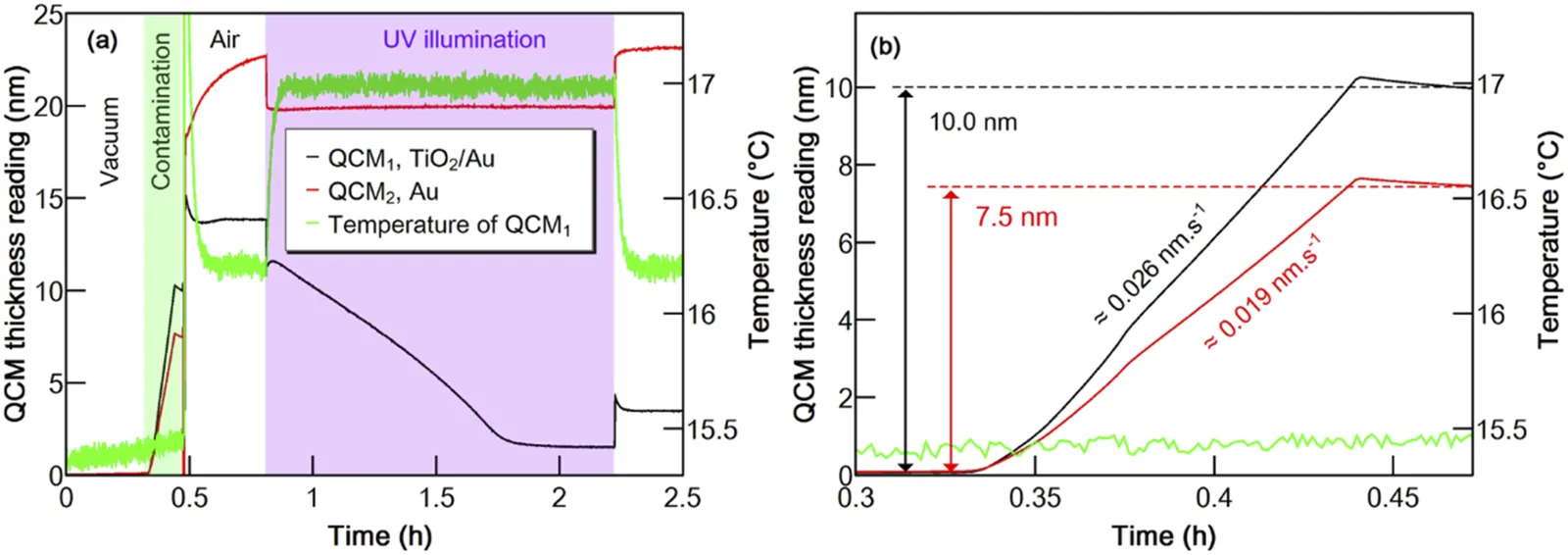
(a) Results of the pumping, contaminating, venting, and illuminating stages of the QCM experiment (b) the zoomed-in view of the contamination stage (Reprinted with permission from ref. 50 Copyright 2020 AIP Publishing.).

Throughout the study, Henry *et al.* used the measurement of thickness of the paraffin film instead of change in mass, based on a mathematical derivation. In the contamination period, QCM_1_ and QCM_2_ acquired 10 nm and 7.5 nm thickened films of paraffin oil, respectively. In the venting phase, these values changed instantly, which was attributed to a transient measurement artifact. The response of QCM_2_ was not affected by UV illumination, whereas QCM_1_ displays a gradual decrease in thickness, which was assigned to the photocatalytic degradation of paraffin oil. The variation of temperature in the vicinity of QCMs was also reported, which shows less than 2 °C change throughout the experiment. They then repeated the QCM experiment under a nitrogen atmosphere to observe completely suppressed photocatalytic degradation, with no change in the thickness of the film. This indicates that the contribution of O_2_ and water to the radical generation is important.

To evaluate this, Henry *et al.*^66^ conducted the same QCM methodology as in the previous study, while varying the thickness of the TiO_2_ coating, thickness of the paraffin oil film, UV irradiance, partial pressure of air, relative humidity, and partial pressure of O_2_. The QCM results were employed to calculate the rate of photocatalytic degradation of paraffin oil. The variation in thickness of the photocatalyst (between 20 nm and 100 nm) did not affect the photocatalytic degradation rate. However, the rate slightly decreased with increasing thickness of the contaminant, which got plateaued after 50 nm of thickness. The rate increased with increasing UV irradiance, as expected. The photocatalytic degradation rate showed a linear increasing trend with the increment of the partial pressure of air, as well as the relative humidity. The increment of partial pressure of O_2_ caused a gradual increase in photocatalytic rate, which reached a plateau after around 120 mbar. Overall, the combined results of the two consecutive studies,^50,66^ obtained using continuous QCM measurements, provide valuable insights into QCM-based photocatalysis and demonstrate a well-controlled experimental design.

#### 4.1.2 Solid, liquid or aqueous pollutants

In 2004, Nakamura *et al.*^35^ used a TiO_2_-coated QCM to monitor the photocatalytic degradation of India ink. In this experiment, they employed a black light which emits UV radiation with 2 mW cm^−2^ irradiance as the light source. They prepared three TiO_2_ coatings with thicknesses of 136 nm, 81 nm, and 58 nm. They also performed calcination of the TiO_2_-coated QCM at temperatures of 200 °C, 250 °C, and 300 °C, while keeping the thickness constant at 80 nm. Each QCM was immersed in India ink for 30 min to adsorb the ink. Subsequently, each dried QCM was irradiated while monitoring the frequency shift at 10 min time intervals.

Being consistent with reports linking calcination with improved TiO_2_ crystallinity and photocatalytic activity,^67,68^ Nakamura *et al.* reported that when the calcination temperature increased from 200 °C to 300 °C, about a three-fold increment in the mass loss was monitored within 1 h irradiation time. Fig. 7 depicts these results.

**Fig. 7:**
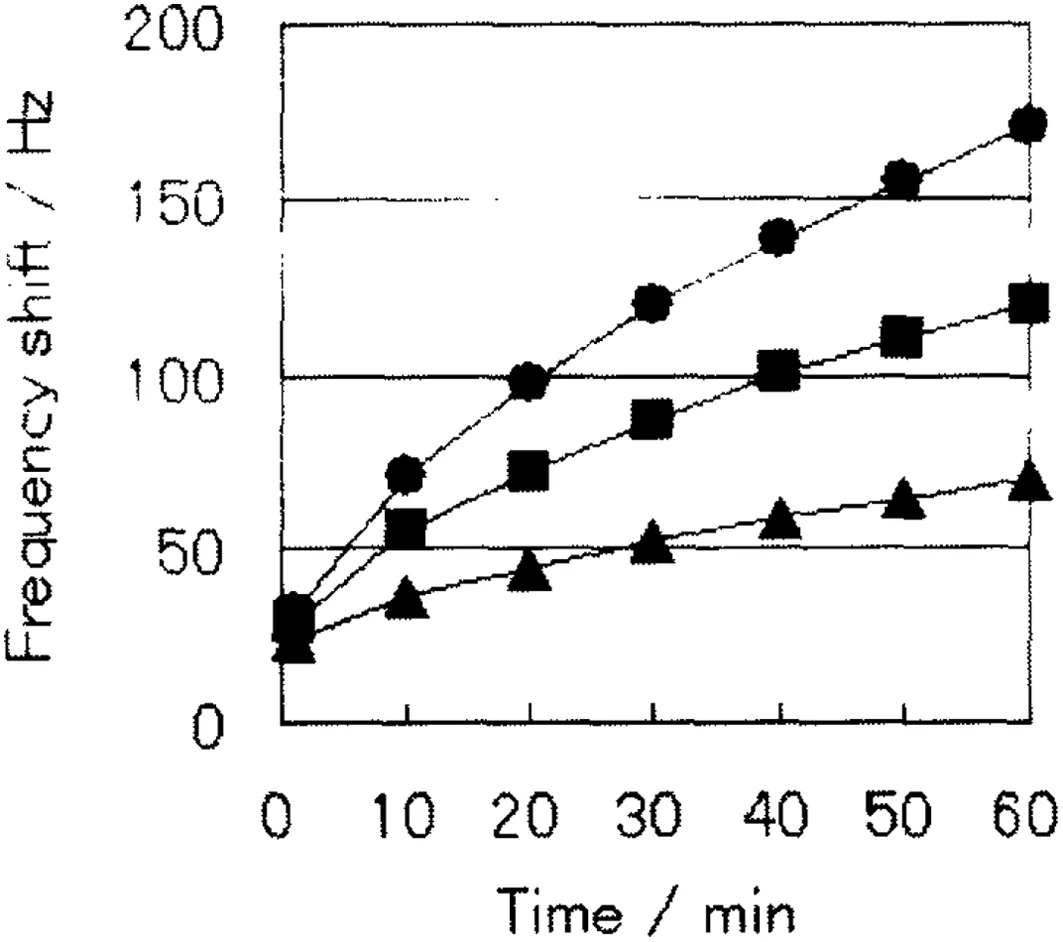
QCM frequency shift in the photocatalytic degradation of India ink on TiO_2_ coating calcined at 200 °C (▲), 250 °C (■), and 300 °C (●). (Reprinted with permission from ref. 35 Copyright 2004 The Authors).

Similar QCM results were shown for the experiments carried out while varying the thickness of the photocatalytic coating. When the thickness increased from 58 nm to 136 nm, the mass loss was tripled. This was attributed to the increasing surface area of the photocatalyst, where more ink molecules could be adsorbed. This explanation was further supported by SEM images. As one of the pioneering applications of QCM to photocatalysis, this study established an important basis for subsequent investigations.

In 2009, Chin *et al.*^34^ conducted a research to study the photocatalytic oxidation of lamp soot using the QCM technique. Initially, they spin-coated the 5 MHz Au QCM surface with TiO_2_ followed by calcination at 350 °C. A hurricane lamp with a 1.8 cm linear flame, fixed next to a custom-made rotating QCM setup, was used to generate soot. By using this setup, lamp soot was directly deposited on the TiO_2_-coated QCM surface. Subsequently, the prepared QCM was irradiated by a 100 W UV spot lamp, while continuously monitoring the QCM frequency variation *in situ* and in real-time. This procedure was repeated for different soot loadings and for different TiO_2_ loadings, which was controlled by the number of passes of QCM through the lamp flame, and the number of TiO_2_ coatings applied, respectively. Fig. 8 depicts the results of this study.

**Fig. 8:**
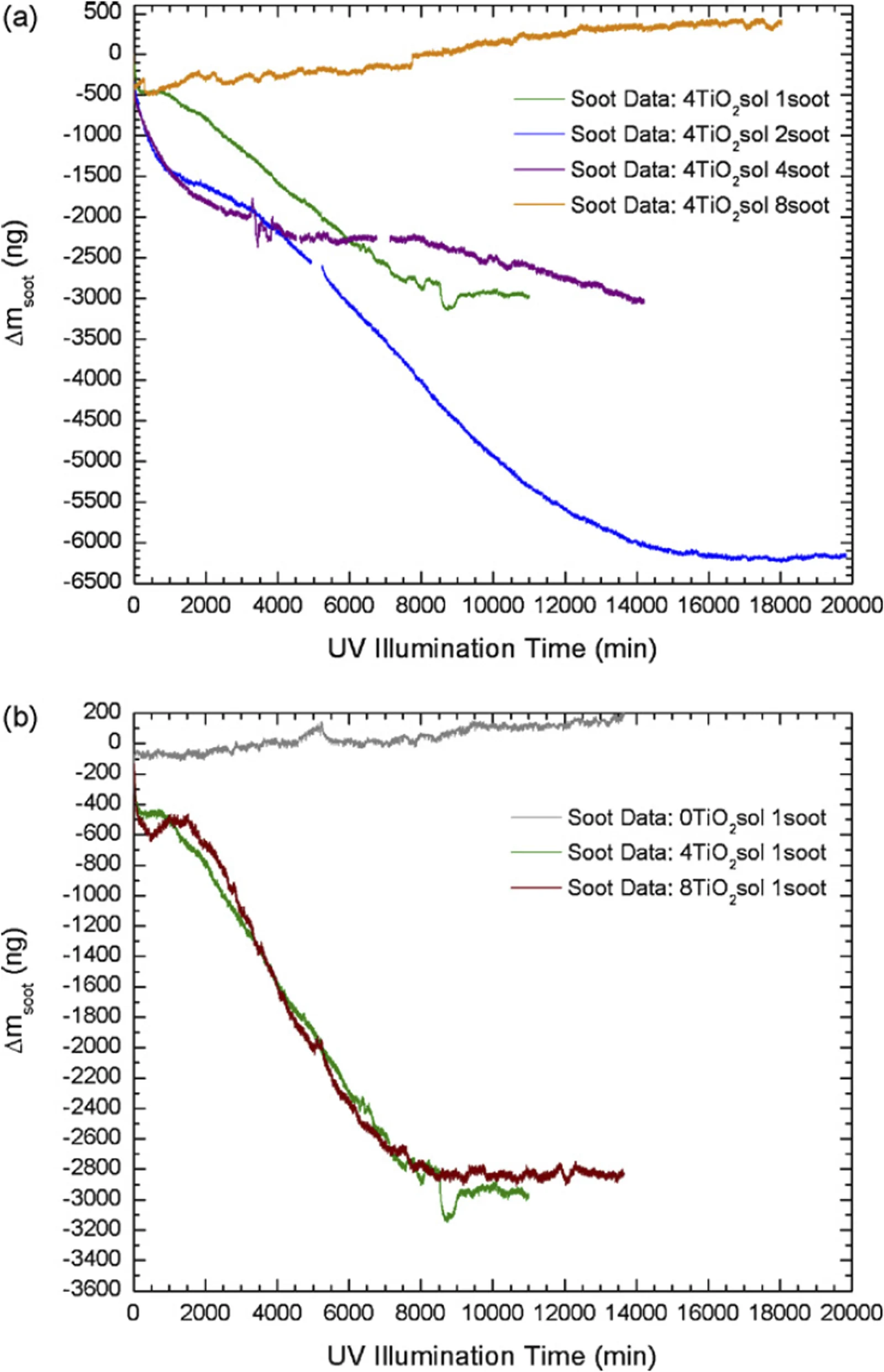
The real-time mass changes occurred in the photocatalytic oxidation of lamp soot to CO_2_, when (a) 1, 2, 4, and 8 loadings of soot was deposited on 4 times TiO_2_-coated QCM (b) soot deposited on bare QCM, 4, and 8 times TiO_2_-coated QCM (Reprinted with permission from ref. 34 Copyright 2009 Elsevier).

It is noteworthy that the data on the *y* axis is usually reported as frequency change, whereas Chin *et al.* calculated and presented the real-time mass change against irradiation time. For once- and twice-soot-loaded QCMs, the initially deposited soot masses were 3000 ng and 6200 ng, respectively. From the results shown in Fig. 8(a), it is clear that these masses were totally removed by photocatalytic oxidation. However, for the higher soot loadings, the soot removal was incomplete. Chin *et al.* explained this result by the screening effect caused by excess soot, making the TiO_2_ surface unreachable for UV photons. This reasoning is validated by other researchers as well.^69–71^ As shown in Fig. 8(b), the number of TiO_2_ coatings which is a measure of the thickness of the catalytic film did not affect the extent or the kinetics of the photocatalytic oxidation of soot. This was explained by the constant catalytic surface area in both cases, as soot was not able to penetrate into the TiO_2_ loading. The QCM data were then fitted into a kinetic model to calculate the rate constants and formal quantum efficiency. This work by Chin *et al.* is significant due to multiple reasons, such as choosing a solid pollutant, extended duration (about 14 days for the longest measurement reported) of continuous QCM monitoring, and non-conventional deposition method of the pollutant.

A modified QCM design was introduced by Abe *et al.*^32^ in 2009. Overall, they constructed a QCM array to minimize environmental impacts on real-time QCM measurements, and then validated it against a standard QCM in monitoring the photocatalytic degradation of methylene blue (MB) dye and sodium dodecylsulfate (SDS). In this study, Abe *et al.* first designed a customized QCM starting from a commercial quartz wafer using photolithography, iron etching, and other experimental techniques. The outcome was a 17 MHz Au QCM with a mass sensitivity of about 60 pg Hz^−1^. This QCM was more sensitive to surface mass changes compared to QCMs used in other studies, such as 5.7 ng Hz^−1 21^ and 1 ng Hz^−1^.^8^ Subsequently, Abe *et al.* constructed a monolithic QCM array by closely placing two of as prepared QCMs at a separation of 3 mm, while each was connected to a separate oscillation circuit. These circuits were then connected to a common frequency counter and then to a computer. When Abe *et al.* illuminated these bare QCMs with UV, they observed a gradual decrease in QCM frequency for a single QCM, whereas this effect was eliminated in the QCM array. As they claimed, this proves that a QCM array could compensate for the environmental changes caused by UV irradiation. These results are shown in Fig. 9(i). However, the said UV-induced effect on bare QCMs was not reported by any other QCM studies presented in this review, probably because their QCM mass sensitivities were significantly larger than that of the custom-made QCM discussed here.

**Fig. 9:**
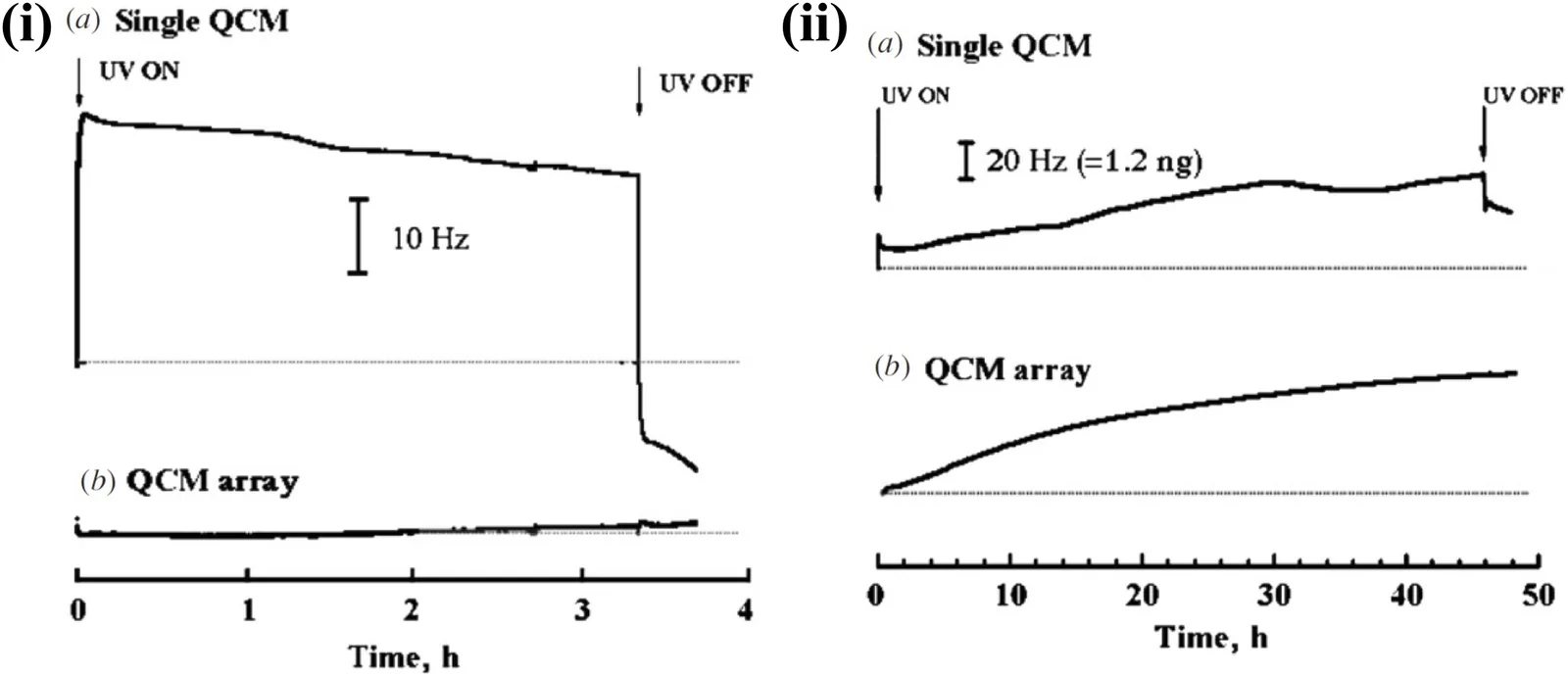
(i) Frequency responses for (a) bare single QCM and (b) bare QCM array under UV irradiation, (ii) Frequency responses for the photocatalytic degradation of SDS coated on (a) TiO_2_-coated single QCM (b) TiO_2_-coated QCM array under UV irradiation (Reprinted with permission from ref. 32 Copyright 2009 Institute of Physics Publishing).

After this background study, both these QCMs in the QCM array were spin-coated with TiO_2_ to achieve uniform coatings. Only one was spin-coated again with MB or SDS while the other QCM remained as a reference. These QCMs were dried, and subsequently, only the QCM with the organic pollutant was exposed to UV irradiation, as previously designed in the construction of the QCM array setup. The photocatalytic degradation was monitored continuously, *in situ* and in real-time, by both single QCM and QCM array methods. The results of photocatalytic degradation of SDS are shown in Fig. 9(ii). It can be observed that the QCM array managed to measure the mass loss more smoothly than expected. Similar results were obtained for MB degradation. Abe *et al.* also conducted a separate QCM study using their QCM array aiming to observe the dependence of the photocatalytic activity of TiO_2_ on annealing temperature. They reported that the same TiO_2_ loading was able to degrade almost two-fold SDS mass when the annealing temperature was increased from 573 K to 623 K. This is in agreement with previous reports.^35,67,68^

The above-discussed monolithic QCM array displayed excellent environment resistance and hence, more reliability on sensitive mass measurements. However, their custom-made QCM array was not adopted by any other QCM photocatalytic assessments reported in this review, probably because of the technological advancement in new built-in QCM setups in the market. Furthermore, executing QCM measurements within a dedicated system under static environmental parameters such as constant temperature obviates the need for environmental baseline compensation. Nevertheless, this work represents an important development in the application of QCM to photocatalytic measurements.

In 2023, Shimosako *et al.*^52^ reported a QCM photocatalytic study dedicated to space applications. This interesting feasibility study involves UV-induced photocatalytic degradation of citric acid under both atmospheric and vacuum conditions. They employed a 5.95 MHz QCM spin-coated with TiO_2_-siloxane binder mixture for the measurements. The reason to use siloxane binder could be better adhesive properties, which must be crucial for applications under vacuum. Citric acid was then deposited on both bare QCM and TiO_2_-coated QCM using the vacuum evaporation technique. Although citric acid provides a convenient model contaminant because of its low desorption under the selected conditions, the use of additional contaminants representative of spacecraft environments would further strengthen the practical relevance of this approach.

In the case of UV irradiation, they used an uncommon approach where they paused the irradiation multiple times. After each UV irradiation, there was a longer time window where QCM was kept under dark conditions before continuing the irradiation. The plateaued mass readings before and after one irradiation cycle were used to calculate the mass loss during the respective irradiation. The experiment was repeated for both contaminated bare QCM and TiO_2_-coated QCM separately under atmospheric and vacuum conditions. The experiment was carried out for longer durations under vacuum (600 h), compared to atmospheric conditions (250 h). Accordingly, the total UV exposure was 200 h for the former, whereas it was limited to 50 h for the latter.

The cumulative mass loss during each irradiation cycle was plotted against irradiation time, as shown in Fig. 10. Similar amounts of citric acid were desorbed from the bare QCM surface both under vacuum and in the atmosphere. These results also show that TiO_2_-coated QCM is able to partially decompose citric acid even under vacuum. A complete degradation, as expected, occurred under atmospheric conditions, owing to the ample presence of water and O_2_ to generate active species. The results of TiO_2_-coated QCM under vacuum were then fitted to an exponential function to calculate the maximum removal of citric acid, which was approximately 2 µg cm^−2^.

**Fig. 10:**
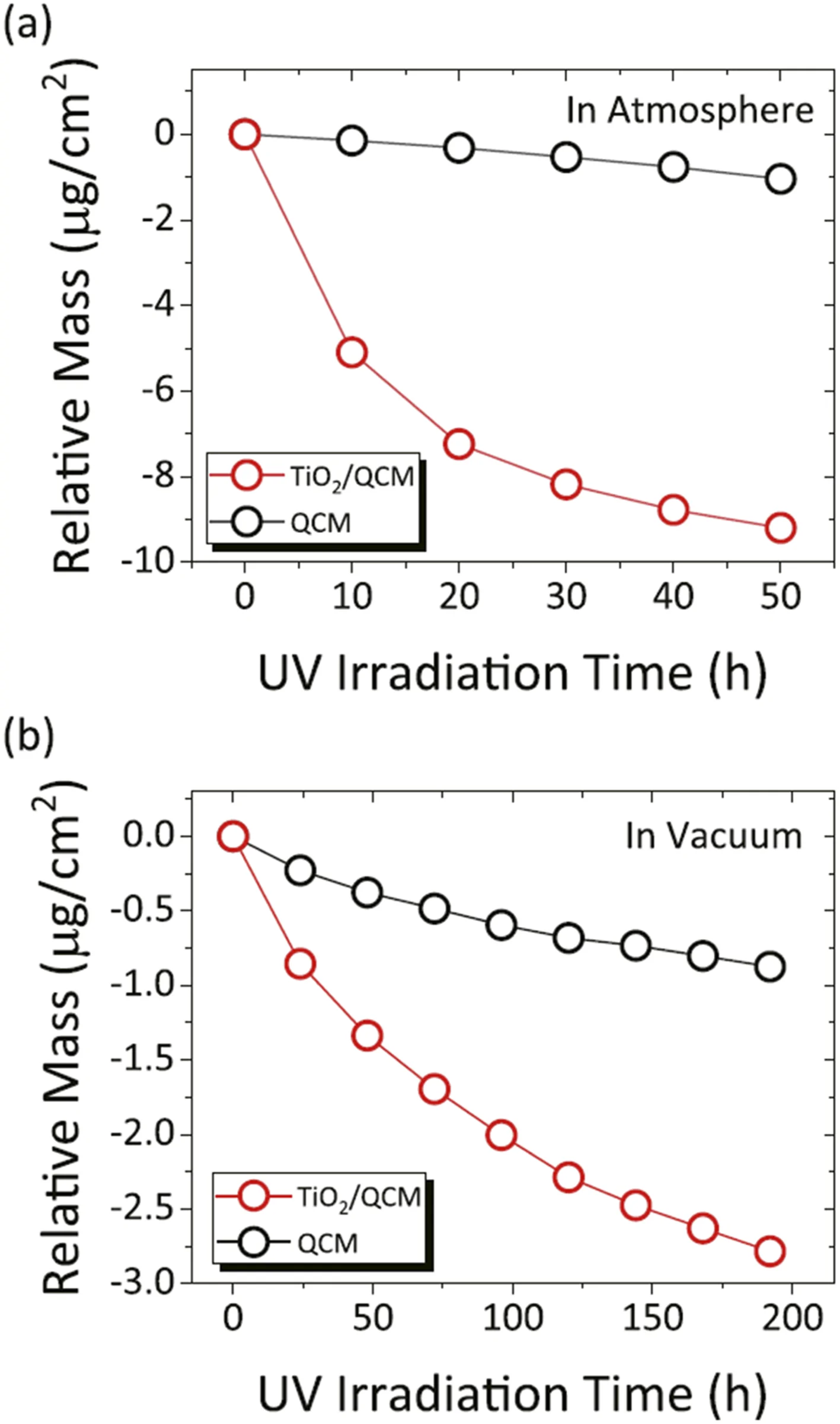
Variation of mass of citric acid coated on QCM and TiO_2_-coated QCM under UV irradiation in the (a) atmosphere (b) vacuum (Reprinted with permission from ref. 52 Copyright 2023 Elsevier).

Continuous irradiation could provide an alternative experimental approach in which the complete QCM response could be fitted directly to an appropriate kinetic model, potentially reducing the number of intermediate calculations. Nevertheless, this study by Shimosako *et al.* shows the versatility of the QCM method for photocatalytic assessments.

Manuda *et al.*^8^ recently reported a typical QCM experiment to validate the photocatalytic performance of TiO_2_ coatings at the solid-air interface. They used a 5 mg ml^−1^ aqueous suspension of P25 commercial TiO_2_ nanopowder to spin-coat 10 MHz Au QCMs. Aqueous methyl orange (MO) was employed as an organic pollutant. The photocatalytic degradation of MO in the presence of UV light was continuously monitored using QCM for a period of 6 h (region 1) until the frequency response was plateaued. Subsequently, while being continuously monitored, the irradiation was paused for 0.5 h (region 2) and then continued for 0.5 h (region 3). This step was carried out to observe the subsequent mass changes after photocatalytic degradation. The entire procedure was repeated for three MO loadings of 55 ng (MO-55), 95 ng (MO-95), and 197 ng (MO-197), to evaluate the dependence of photocatalytic activity on the surface concentration of MO. For all three MO loadings, the mass of TiO_2_ on QCM was kept constant at 3000 ng. The same procedure was repeated on an as-coated QCM without MO to observe any background effects.

In region 1, for MO-197, the mass loss is lower than expected (121 ng), which was assigned to the screening effect caused by the excess presence of MO. The negative effect of higher concentrations of organic pollutants on photocatalytic degradation is also noticed and reasoned in other photocatalytic assessments.^69–73^ Moreover, Manuda *et al.* presented XPS and Raman evidence to prove the presence of MO in MO-197 QCM after photocatalytic degradation. Surprisingly, for both MO-55 and MO-95, the mass loss in region 1 exceeded the initially deposited mass of MO by about 20 ng. This is assigned to the loss of surface water followed by the complete degradation of MO. Since the only TiO_2_-coated QCM also exhibited a similar mass loss of about 20 ng in region 1, this conclusion is further supported. The QCM responses observed in regions 2 and 3 in all cases are attributed to the adsorption and photocatalytic removal of atmospheric water, respectively. Since relative humidity (RH) is a measure of atmospheric water, they carried out another photocatalytic experiment with only TiO_2_-coated QCM under different RH levels (5%, 16%, 45%, 69%). The results show that the same trend is repeated with higher values at higher RH levels, proving the involvement of RH in the photocatalytic system. These results are shown in Fig. 11.

**Fig. 11:**
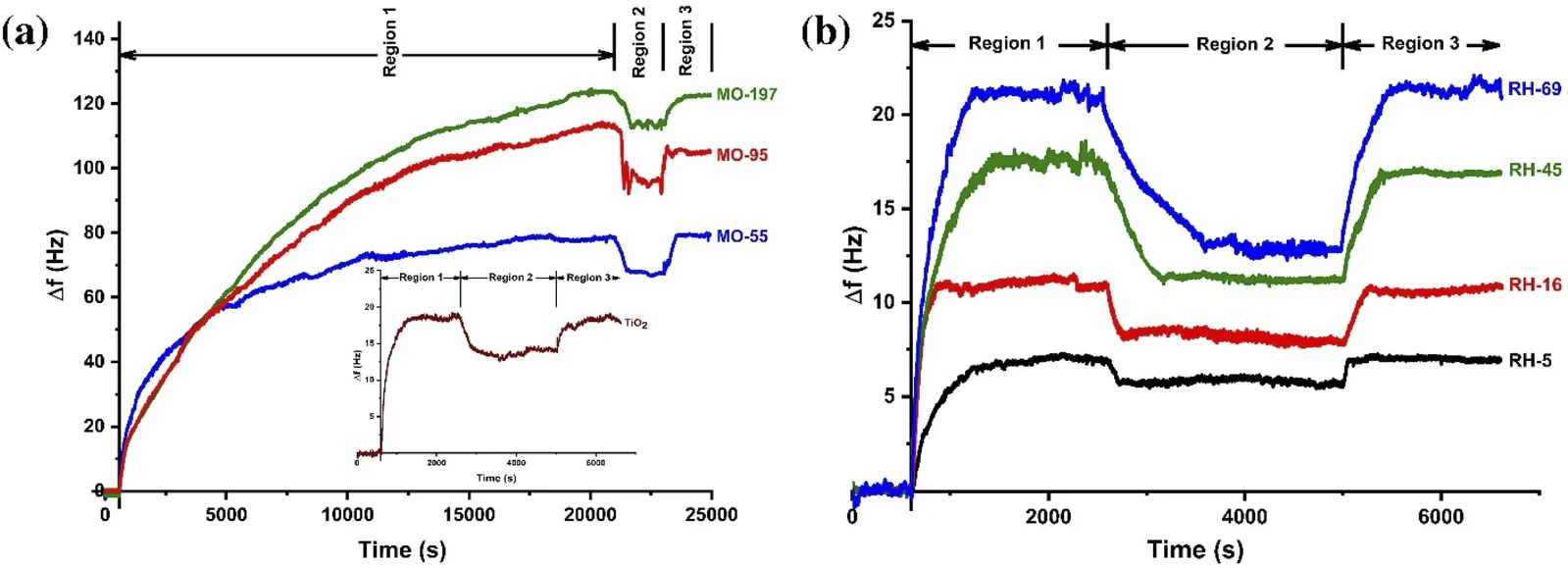
(a) QCM frequency shift of MO-55, MO-95, and MO-197 at 60% RH. Inset shows the frequency shift of only TiO_2_-coated QCM at 60% RH. (b) QCM frequency shift of only TiO_2_-coated QCM at the RH levels of 5%, 16%, 45%, and 69% (Reprinted with permission from ref. 8 Copyright 2024 Royal Society of Chemistry).

The conclusions of this study include the liberation of radicals from the surface, as a result of UV irradiation. To justify the statement, they compared the QCM response under UV and visible light illuminations on only TiO_2_-coated QCM. Under visible light, there is no significant frequency shift, which they claimed due to the inability of TiO_2_ to generate electron–hole pairs, where no radical is formed. This interpretation is consistent with the bandgap of P25 being approximately 3.2 eV, where an electron can only be promoted by UV or higher energies.^59^ Thus, this study of Manuda *et al.* has not only pointed out the importance of RH for photocatalytic coatings but also identified a new radical detection possibility based on QCM, at least applicable to the area of photocatalysis.

In 2025, Manuda *et al.*^74^ reported another QCM-based photocatalytic assessment as a continuation of the above study. In this study, they synthesized a nanocomposite with reduced graphene oxide (rGO) and P25 to fabricate the 10 MHz Au QCMs. As in the first study, MO was used as the organic pollutant. Even though the photocatalytic nanocomposite with a bandgap of 2.84 eV was determined to be visible light-active, the *in situ* real-time QCM data reflected a partial degradation of MO under visible irradiation. However, MO was completely degraded under UV irradiation. Fig. 12 elaborates the QCM response in these continuous, cumulative irradiations. Mass gains are observed under dark conditions, similar to the ones detected and explained in their previous study.^8^ The anomalous QCM response during photocatalytic degradation of MO under the two irradiation conditions led Manuda *et al.* to investigate its underlying cause, which was attributed to a wavelength-dependent mechanistic change. They strengthened this conclusion with a series of UV-Visible spectroscopic measurements. By interpreting these measurements, the partial mass loss under visible irradiation is explained as the formation of stable degradation intermediates, which are prone to degrade under UV irradiation.^74^

**Fig. 12:**
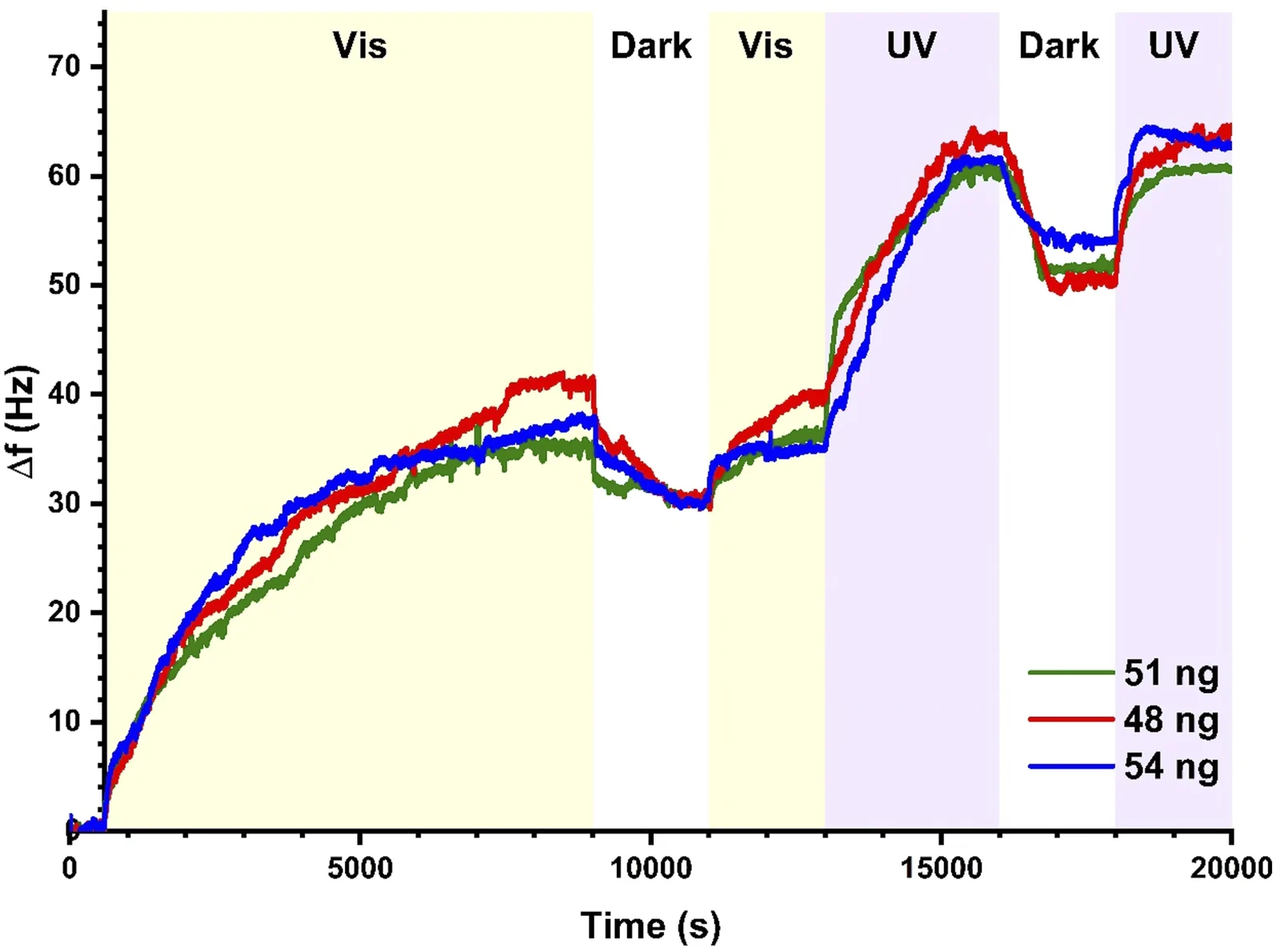
QCM frequency shift under continuous visible (Vis) and UV irradiation indicating photocatalytic degradation of MO (Reprinted with permission from ref. 74 Copyright 2025 The Authors).

### 4.2 Solid–liquid interface

Yang *et al.* reported three QCM-based studies on the photocatalytic degradation of bilirubin in 2008,^48^ 2009,^21^ and 2011.^49^ Because of the methodological and empirical similarities among these three studies, the comprehensive 2009 investigation was selected as the baseline for this discussion. The primary difference across the literature is the specific photocatalytic coating employed. Consequently, novel insights or distinct findings from the remaining two articles are briefly addressed at the end of the discussion.

In this study,^21^ Yang *et al.* evaluated the photocatalytic removal of bilirubin using nanostructural hydroxyapatite (HA). Since bilirubin is a biological metabolite that travels through the human bloodstream, the temperature and pH of this study were maintained at 37 °C and 7.4, respectively, to mimic the actual human body conditions. QCM method was used *in situ* and in real-time to continuously monitor both the adsorption of bilirubin on HA and the photocatalytic degradation of bilirubin. Nano HA was spin-coated onto a 9 MHz Au QCM, which was then heat-treated at 400 °C. The experimental cell setup only allowed to the coated side of the QCM to be in contact with the bilirubin solution. After mounting the as-coated QCM, they allowed different concentrations of bilirubin, ranging from 0.2 µM to 5.0 µM, to enter into the cell to facilitate the adsorption. Subsequently, the dried QCM *ex situ* results suggested that 1.0 µM concentration is the surface saturation limit. Therefore, for *in situ* continuous monitoring, they employed 1.0 µM bilirubin.

In the beginning of the *in situ* adsorption study, DI water was introduced into the cell until the QCM frequency response was settled. Then, within a period of 5 s, bilirubin was introduced. This procedure is important because if the QCM frequency is not settled by the moment when bilirubin is injected, the adsorption cannot be monitored due to excessive damping that occurs specifically at the solid–liquid interface. The results of this experiment are depicted in Fig. 13(a). According to the results, QCM without the HA coating does not adsorb bilirubin, while HA adsorbs more bilirubin when the concentration is increased. However, after fitting the data into the pseudo-first-order kinetic model, they found that the calculated adsorption–desorption equilibrium constants for the two concentrations are almost identical. Hence, Yang *et al.* concluded that the interaction between bilirubin and HA does not depend on the bilirubin concentration.

**Fig. 13:**
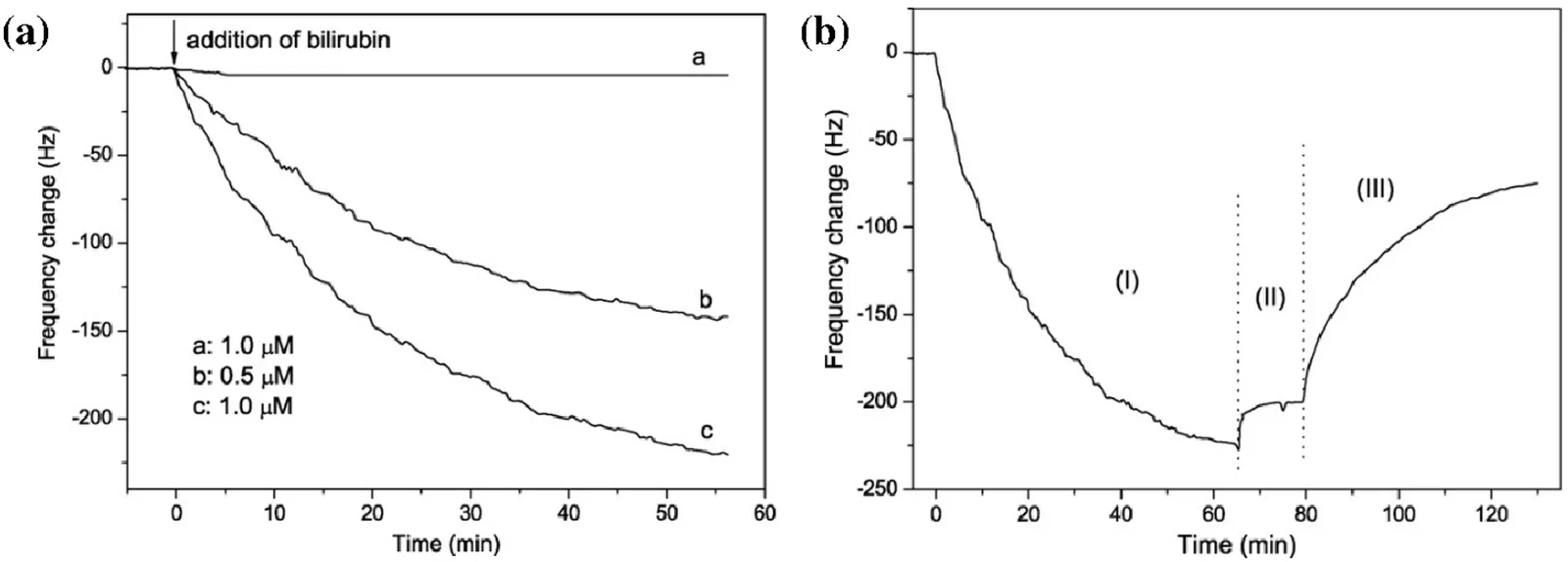
(a) QCM frequency shift after the addition of bilirubin to the detector cell of (a) bare QCM; (b) with HA coating at 0.5 µM bilirubin concentration (c) with HA coating at 1.0 µM bilirubin concentration (b) QCM frequency shift of (I) adsorption of bilirubin, (II) rinsing with DI water, (III) photocatalytic degradation of 1.0 µM bilirubin (Reprinted with permission from ref. 21 Copyright 2009 Elsevier).

The photocatalytic experiment was conducted after exposing to 1.0 µM bilirubin. Fig. 13(b) shows the QCM results of this experiment. Region I shows the adsorption, while region II shows an interesting step of rinsing with DI water. This allows the weakly bound bilirubin molecules to desorb from the surface, which could be observed by the small mass loss in the region. It is noteworthy that after this rapid mass loss, the QCM reading becomes more stable than at the end of region I. Finally, region III shows the photocatalytic degradation of bilirubin under UV irradiation. A complete degradation was not observed by Yang *et al.* Nevertheless, a substantial degradation rate was observed.

As stated earlier, the other two publications on bilirubin degradation by Yang *et al.* incorporated molecularly imprinted TiO_2_,^49^ and HA-modified nanocrystalline TiO_2_ (ref. 48) as photocatalysts. The QCM operating procedure is the same as discussed above. In both studies, the adsorption kinetics are slower than above. However, complete photocatalytic degradation of the adsorbed bilirubin was achieved in both studies within a similar time interval. This suggests that the incorporation of TiO_2_ into the coating increases the photocatalytic activity against bilirubin.

Hidaka *et al.* studied the adsorption and photocatalytic degradation of phenol and catechol using the QCM method in 2003.^22^ This work was extended for benzoic acid and salicylic acid in 2006.^20^ Since both publications have similar methodologies, only the first study is discussed here in detail. The key findings of the second study are briefly discussed later.

In the first study, they dip-coated a 6 MHz Pt QCM with TiO_2_ as the photocatalyst. Subsequently, the adsorption of water onto a bare QCM and a TiO_2_-coated QCM under UV irradiation and at different pH levels was monitored continuously, *in situ* and in real-time, at the solid–liquid interface. They concluded that water adsorption was more significant under UV irradiation than under dark conditions, which was explained by photoinduced chemisorption of water. They also showed that water adsorption is the highest at pH 7, compared to pH 5 and pH 9. Subsequently, they checked for the optimal concentrations of phenol and catechol for the best adsorption on TiO_2_-coated QCM, under the mentioned three pH levels. The *ex situ* QCM results showed that for concentrations higher than 0.007 mM, the surface was saturated in 20 min, while the best adsorption characteristics were displayed at pH 7. Therefore, the subsequent photocatalytic experiments were conducted at the selected phenol or catechol concentration of 0.01 mM.

In the photocatalytic degradation process, they exposed the TiO_2_-coated QCM to 0.01 mM solutions of phenol and catechol, under UV irradiation from the beginning. This method does not yield accurate QCM measurements on photocatalytic degradation, as processes such as photoinduced adsorption and photoinduced desorption may take place simultaneously. Hidaka *et al.* had also noticed this. They also presented the variation of concentration, detected by fluorimetric analysis, against the irradiation time. According to these graphs, the concentrations of phenol and catechol decreased by nearly 20% and 50%, respectively. Hidaka *et al.* assigned this decrement directly to photocatalytic degradation, which is arguable. The concentration could be decreased due to adsorption (irrespective of photoinduced or general) as well since the TiO_2_ surface was not saturated before the irradiation commenced. Consequently, these methodological limitations precluded the effective utilization of the QCM for acquiring rigorous quantitative data regarding photocatalytic degradation kinetics. However, this study can be considered one of the pioneering applications of QCM to photocatalysis.

As introduced earlier, Hidaka *et al.* repeated the same methodology in 2006 for benzoic acid and salicylic acid.^20^ Since the overall procedure was the same in every experimental aspect (QCM type, experimental setup, photocatalyst, pH levels, ideal concentration of 0.01 mM, and fluorimetric determination of concentration changes), except for the pollutants, only the continuous QCM readings of photocatalytic degradation are discussed here. The experimental outcomes are illustrated in Fig. 14, where a recurring methodological limitation from the study by Hidaka *et al.* is observed. The concurrent occurrence of photocatalytic degradation and alternative photoinduced phenomena confounds the analysis of the net mass loss. To isolate these mechanisms, the TiO_2_ surface should have been pre-saturated prior to the initiation of UV irradiation.

**Fig. 14:**
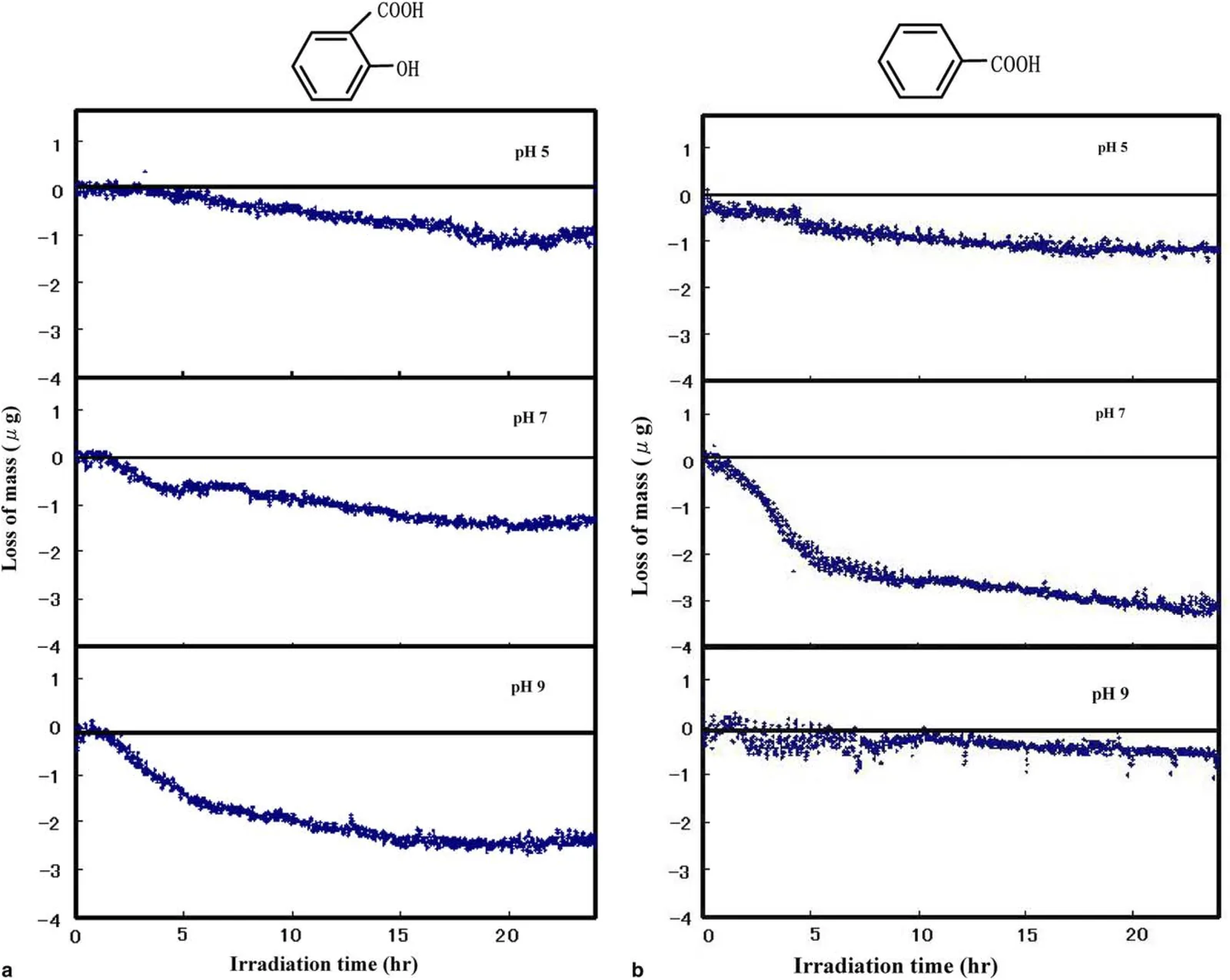
Change in mass against irradiation time in the photocatalytic degradation of (a) salicylic acid and (b) benzoic acid (Reprinted with permission from ref. 20 Copyright 2006 Elsevier).

In 2007, another photocatalytic assessment was carried out by Hidaka *et al.* to study the photoreduction of Ag^+^ on the TiO_2_ surface.^75^ As a result of the photocatalytic reduction of Ag^+^, Ag clusters are deposited on the surface. QCM technique was involved in measuring the mass deposited. They employed a 6 MHz Pt QCM, dip-coated with TiO_2_ followed by heat treatment at 350 °C, for this research. An as-coated QCM was allowed to equilibrate with an aqueous 0.1 mM AgNO_3_ solution under dark conditions. Subsequently, it was illuminated by a UV laser under 0.64 mW cm^−2^ irradiance, while continuously monitoring the QCM response *in situ* and in real-time. The irradiation was halted for around 25 min to observe the mass deposition behavior. Fig. 15 shows the results of this study. Hidaka *et al.* plotted the graph with deposited Ag mass on the *y*-axis, which is usually dedicated to the frequency change (Δ*f*). From the results, it is obvious that photocatalytic reduction of Ag^+^ and subsequent deposition of Ag occurs under UV irradiation, and it takes place only on the surface of TiO_2_, which is the photocatalyst. The deposited Ag mass before the irradiation halt is higher than what is observed after the halt. However, the latter shows better kinetics. Hidaka *et al.* did not fully elucidate these variations in mass deposition, noting that further spectroscopic analysis is required for a definitive explanation. Nonetheless, the authors hypothesized that this phenomenon correlates with the size distribution of the formed metal clusters, specifically involving Ag and Ag^2+^ species.

**Fig. 15:**
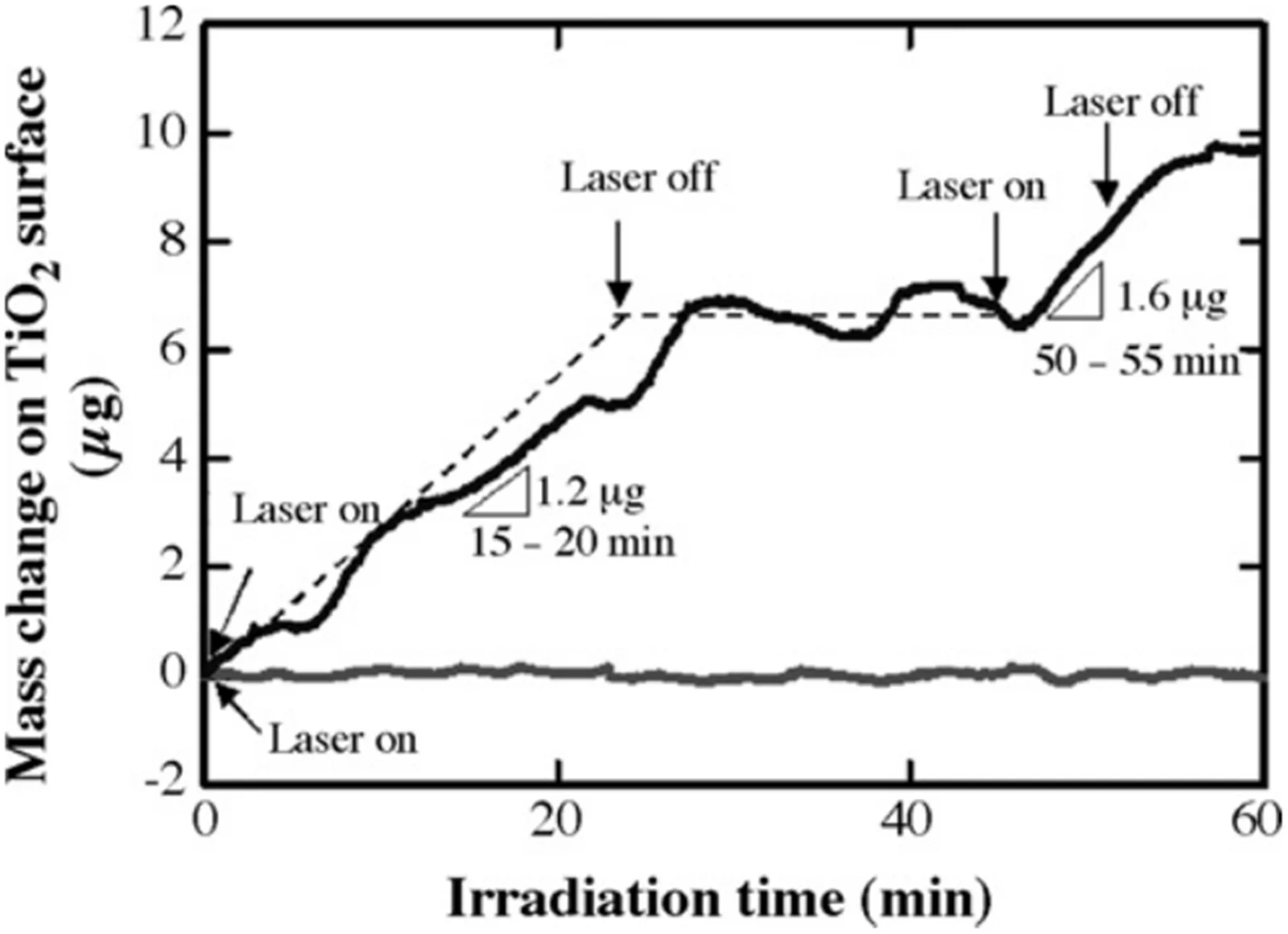
The photocatalytic Ag^+^ reduction and subsequent Ag deposition under UV irradiation on a TiO_2_-coated QCM surface (top curve), and on a bare QCM surface (bottom curve) (Reprinted with permission from ref. 75 Copyright 2007 Elsevier).

In the same study, they investigated the dependence of photocatalytic mass deposition on pH. They showed that at pH 6, the deposition was almost 3-fold compared to that at pH 4, explained by the surface charge difference of TiO_2_. Hidaka *et al.* also tested the effect of hole scavengers in the photocatalytic reduction of Ag^+^. If a hole scavenger is present, less recombination of photogenerated electron–hole pairs occurs,^76^ which increases the probability of Ag^+^ reduction. Methanol and ethanol were used as the hole scavengers in this study to observe significantly greater Ag depositions compared to the previous, at pH 6. The extent of deposition is the highest in methanol, explained by its ample presence on the TiO_2_ surface due to its smaller size than ethanol. Furthermore, as a countermeasure to the argument that this QCM mass gain occurred due to the adsorption of formaldehyde and acetaldehyde formed during the scavenger process of methanol and ethanol, respectively, Hidaka *et al.* carried out a separate adsorption study with formaldehyde and acetaldehyde. The results of this supplementary QCM study, which was carried out under the same conditions as the main scavenger study, showed that the adsorbed mass of these species has a negligible contribution to the QCM readings of the main study. Overall, this work by Hidaka *et al.* is a descriptive illustration of what the QCM method is capable of monitoring in photocatalysis.

Kallio *et al.*^77^ employed QCM-D to study the photocatalysis of TiO_2_ against several fatty acids and other wood extractives. QCM-D is particularly useful for examining materials that form soft or weakly coupled layers on the QCM surface. In this case, frictional losses result in the damping of oscillations, characterized by a decay rate of the amplitude that depends on the viscoelastic properties of the adsorbed material. Using QCM-D, changes in the dissipation factor, denoted as Δ*D* = *D* − *D*_0_, can be quantified, where *D*_0_ = dissipation factor of the blank QCM immersed in the solvent, and *D* = dissipation factor after material adsorption.

In this comprehensive study, TiO_2_ films were grown on QCM using the atomic layer deposition (ALD) technique. The organic substances were coated on the as prepared QCMs by either Langmuir–Blodgett layer deposition or by spin-coating technique. Subsequently, photocatalytic degradation was continuously monitored at both solid-air and solid–liquid interfaces, *in situ* and in real-time. For the solid–liquid interface, before commencing the UV irradiation, the as-coated QCMs were immersed in water to equilibrate. Fig. 16(a) shows the QCM-D response in the degradation of β-sitosterol with Langmuir–Blodgett multilayers, while Fig. 16(b) shows the QCM-D response in the degradation of a spin-coated wood resin. By comparing the magnitude of detachment before irradiation, it is clearly seen that Langmuir–Blodgett deposition has better adhesive properties compared to spin-coating. This is also proven by the larger Δ*D* value obtained for the wood resin during photocatalytic degradation, in which case degraded products are readily transported to water. Under UV irradiation, a typical mass loss followed by a plateauing effect can be seen in both graphs. The wood resin has faster degradation kinetics.

**Fig. 16:**
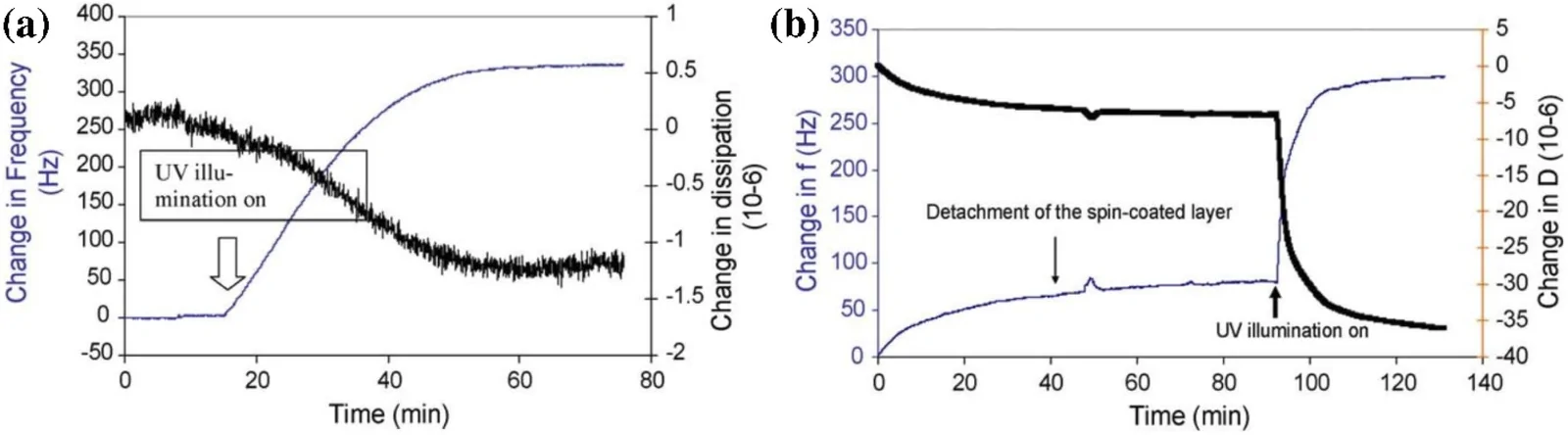
QCM-D response in the photocatalytic degradation of (a) β-sitosterol (b) a wood resin (Reprinted with permission from ref. 77 Copyright 2006 Elsevier).

This work by Kallio *et al.* presents detailed QCM analyses of photocatalytic degradation of stearic acid, oleic acid, dehydroabietic acid, capric acid, linoleic acid, β-sitosterol, and a wood resin while formulating a degradation mechanism to satisfactorily explain each experimental result. Gathering all these aspects exceeds the scope of this review. Even though the degraded organic substances were deposited on the catalytic surface, this work is categorized under Section 4.2 because of the involvement of water.

The studies discussed are summarized in Table 1, in the order of appearance.

**Table 1:** A summary of QCM assessments on photocatalysis discussed in this article

QCM configuration	Photocatalyst	Coating method	Pollutant	Photocatalytic process monitoring	Irradiation	Published year	Reference
**Solid-air interface: gaseous pollutants**							
6 MHz, Pt	TiO_2_	Spin-coating	Acetaldehyde, toluene	*In situ,* intermittent	UV	2004	35
10 MHz, Au	ZnO	Direct growing	Hexanethiol	*In situ,* intermittent	UV	2009	17
6 MHz, Au	TiO_2_/ZnO	Dip-coating	Acetone	*In situ,* intermittent	UV	2015	47
6 MHz, Cr/Au	TiO_2_	Atomic layer deposition	Paraffin	*In situ,* continuous	UV	2020	50
6 MHz, Cr/Au	TiO_2_	Atomic layer deposition	Paraffin	*In situ,* continuous	UV	2021	66

**Solid-air interface: solid, liquid or aqueous pollutants**							
6 MHz, Pt	TiO_2_	Spin-coating	India ink	*In situ,* intermittent	UV	2004	35
5 MHz, Au	TiO_2_	Spin-coating	Soot	*In situ,* continuous	UV	2009	34
17 MHz, Au	TiO_2_	Spin-coating	MB, SDS	*In situ,* continuous	UV	2009	32
5.95 MHz	TiO_2_-siloxane binder mixture	Spin-coating	Citric acid	*Ex situ,* intermittent	UV	2023	52
10 MHz, Au	TiO_2_	Spin-coating	MO	*In situ,* continuous	UV	2024	8
10 MHz, Au	rGO-TiO_2_	Spin-coating	MO	*In situ,* continuous	Visible, UV	2025	74

**Solid-liquid interface**							
9 MHz, Au	HA	Spin-coating	Bilirubin	*In situ,* continuous	UV	2009	21
9 MHz, Au	HA-modified TiO_2_	Spin-coating	Bilirubin	*In situ,* continuous	UV	2008	48
9 MHz, Au	TiO_2_	Dip-coating	Bilirubin	*In situ,* continuous	UV	2011	49
6 MHz, Pt	TiO_2_	Dip-coating	Phenol, catechol	*In situ,* continuous	UV	2003	22
6 MHz, Pt	TiO_2_	Dip-coating	Benzoic acid, salicylic acid	*In situ,* continuous	UV	2006	20
6 MHz, Pt	TiO_2_	Dip-coating	Ag^+^	*In situ,* continuous	UV	2007	75
15 MHz, QCM-D	TiO_2_	Atomic layer deposition	Stearic acid, oleic acid, dehydroabietic acid, capric acid, linoleic acid, β-sitosterol, wood resin	*In situ,* continuous	UV	2006	77

## 5. Comparative and critical analysis of QCM-based photocatalytic assessments

The studies discussed above demonstrate that QCM can provide direct, real-time information on mass changes occurring at photocatalyst interfaces during irradiation. However, the reported studies differ substantially in terms of QCM configuration, photocatalyst composition and loading, pollutant identity and loading, irradiation conditions, reaction environment, and data-analysis procedures. Consequently, direct comparison of photocatalytic performance between different studies is not always straightforward. A meaningful evaluation therefore requires consideration not only of the magnitude and rate of the measured mass change, but also of the experimental conditions and the extent to which possible non-photocatalytic contributions to the QCM response have been controlled.

### 5.1 Quantitative comparison of reported photocatalytic performance

A quantitative comparison of the principal QCM-based photocatalytic studies is presented in Table 2. The reported parameters include pollutant loading, photocatalytic mass removal, rate constants or mass-removal rates, photocatalyst thickness, irradiation conditions, and other relevant experimental parameters where these were explicitly reported by the original authors.

**Table 2:** Quantitative parameters reported for selected QCM-based photocatalytic studies

Reference	Photocatalyst	Pollutant	Initial pollutant loading	Observed photocatalytic response	Rate/kinetic parameter	Other quantitative information
35	TiO_2_	India ink	Not reported	Mass loss increased ∼3× when TiO_2_ thickness increased from 58 to 136 nm	Not reported	UV irradiance = 2 mW cm^−2^; TiO_2_ thickness = 58, 81, 136 nm
34	TiO_2_	Lamp soot	∼3000 ng (1 pass)	∼100% oxidation	Model fitted using (*k*_1_, *k*_2_, *k*_3_)	80 nm TiO_2_; 365 nm UV; 1 pass
			∼6200 ng (2 passes)	∼100% oxidation	Model fitted using (*k*_1_, *k*_2_, *k*_3_)	80 nm TiO_2_
			4 passes	∼55% oxidation at 14 000 min	Model fitted	UV transmission strongly reduced
			8 passes	Negligible oxidation	∼0.25% predicted after 20 000 min	∼1% UV transmission through soot
32	TiO_2_	MB, SDS	Not reported	Continuous mass-loss monitoring	Not reported	17 MHz Au QCM; ∼60 pg Hz^−1^ sensitivity
50	ALD-TiO_2_	Paraffin oil	10 nm effective thickness	Complete paraffin film removal in ∼1 h	∼9.2 nm h^−1^	20 nm TiO_2_; reference QCM
66	ALD-TiO_2_	Paraffin oil	10 nm	Complete paraffin film removal in <2 h	Zero-order kinetics; ±10% uncertainty	TiO_2_ = 21–77 nm; irradiance exponent *α* ≈ 0.5
			Variable	Rate decreases with contaminant thickness	Rate plateaus above ∼20–50 nm	O_2_ effect becomes weak at high partial pressure
48	TiO_2_	Bilirubin	1.0 µM	Rate increases to optimum at 0.7 wt% HAP	0.35–1.18 × 10^−3^ s^−1^	37 °C; pH 7.4; *λ* = 280 nm
	0.7 wt% HAP-TiO_2_			Highest reported rate in series	1.18 ± 0.12 × 10^−3^ s^−1^	Adsorbed bilirubin = 34.5 ± 1.45 mg g^−1^
	1.0 wt% HAP-TiO_2_			Activity decreases	0.46 ± 0.05 × 10^−3^ s^−1^	Adsorbed bilirubin = 36.7 ± 1.72 mg g^−1^
22	TiO_2_	Phenol	0.01 mM	Solution concentration decreased ∼20%	Not reported	pH 5, 7, 9 examined
		Catechol		Solution concentration decreased ∼50%		pH 5, 7, 9 examined
75	TiO_2_	Ag^+^	0.1 mM AgNO_3_	Photoreductive Ag deposition	Not reported	UV irradiance = 0.64 mW cm^−2^; deposition ∼3× greater at pH 6 than pH 4
52	TiO_2_-siloxane	Citric acid	Not reported	Partial degradation under vacuum; complete in atmosphere	Maximum removal ≈2 µg cm^−2^ under vacuum	Vacuum test ≈600 h; atmospheric ≈250 h
8	TiO_2_ P25	MO	55, 95, 197 ng	MO-197: 121 ng mass loss; lower loadings showed complete MO mass removal plus ∼20 ng background response	Not reported	TiO_2_ loading = 3000 ng; 60% RH
74	rGO-TiO_2_	MO	51 ± 3 ng	Partial MO mass removal under visible light; complete under UV	Average initial rate (AIR) = 1.86 × 10^−2^ ng s^−1^ initially; 9.43 × 10^−3^ ng s^−1^ after restart	Bandgap = 2.84 eV

An important observation from Table 2 is that the literature does not employ a single universal parameter for describing photocatalytic activity. For example, Yang *et al.* described the degradation of bilirubin using apparent first-order rate constants,^48^ whereas Henry *et al.* quantified the removal of a paraffin layer through changes in film thickness with time.^50^ Chin *et al.* used a kinetic model incorporating several rate constants to describe the oxidation of soot,^34^ while several other studies primarily reported the QCM frequency or mass change as a function of irradiation time.^8,66,74^ Therefore, the numerical values reported in these studies should not be interpreted as directly comparable photocatalytic rate constants.

Nakamura *et al.* demonstrated that increasing the TiO_2_ coating thickness from approximately 58 to 136 nm increased the observed mass loss during photocatalytic degradation of deposited India ink by approximately three times.^35^ This observation suggests that increasing the amount of photocatalyst can increase the number of available active sites under conditions where the photocatalyst loading is initially insufficient. However, this trend is not universal. Henry *et al.* reported that increasing the TiO_2_ thickness over the investigated range did not produce a proportional increase in the paraffin-removal rate.^66^ Similarly, Chin *et al.* found that increasing the TiO_2_ coating thickness did not substantially enhance soot oxidation under their experimental conditions.^34^ These apparently different observations emphasize that photocatalyst thickness cannot be considered an independent measure of photocatalytic activity. Light penetration, pollutant accessibility, film morphology, mass transport, and the optical properties of the pollutant layer may all influence the observed QCM response.

Pollutant loading is another important parameter. The experiments of Chin *et al.*^34^ showed that soot loading strongly influenced the observed degradation behaviour. At relatively low loading, almost complete oxidation could be achieved, whereas increasing the number of soot passes progressively reduced the apparent degradation efficiency. At high soot loading, the pollutant layer substantially attenuated the incident UV radiation, thereby limiting the irradiation of the underlying TiO_2_ surface. This demonstrates that a large initial QCM mass does not necessarily correspond to a proportionally larger measurable photocatalytic mass loss.

A similar distinction between deposited pollutant mass and measured QCM mass change was observed by Manuda *et al.*^74^ During the photocatalytic degradation of methyl orange on TiO_2_-coated QCMs, the initial mass loss for some pollutant loadings exceeded the amount of methyl orange deposited on the crystal. Control experiments using TiO_2_ without methyl orange demonstrated a comparable mass response, indicating that removal of adsorbed surface water contributed significantly to the observed frequency shift. These observations highlight the importance of distinguishing the mass of the target pollutant from changes in the mass of other species present at the photocatalyst surface.

The aqueous studies provide additional evidence that QCM-derived photocatalytic rates depend strongly on the interfacial environment. Yang *et al.* observed that incorporation of hydroxyapatite into TiO_2_ influenced both bilirubin adsorption and photocatalytic degradation, with an optimum composition occurring at an intermediate hydroxyapatite content.^48^ The highest degradation rate was not simply associated with the highest adsorption capacity, demonstrating that adsorption and photocatalytic reaction are related but distinct processes. Excessive adsorption may increase the amount of pollutant accumulated at the interface while simultaneously modifying light penetration, active-site accessibility, or charge–transfer processes.

Therefore, QCM measurements are particularly valuable because they can provide information on the interfacial mass balance. However, the resulting mass loss rate should not automatically be interpreted as an intrinsic photocatalytic activity parameter. A QCM-derived rate represents the combined outcome of adsorption, desorption, surface reaction, mass transport, and, depending on the experimental configuration, environmental changes occurring at or near the sensing surface.

### 5.2 Influence of photocatalyst loading and film thickness

Photocatalyst loading is one of the most important parameters influencing QCM-based measurements because the photocatalyst must provide sufficient active surface area while simultaneously permitting effective irradiation and mass transport. The available literature does not demonstrate a simple monotonic relationship between photocatalyst thickness and photocatalytic mass removal.

In the study of Nakamura *et al.*,^35^ increasing TiO_2_ film thickness resulted in a substantial increase in the observed mass loss of deposited India ink. This behavior is consistent with an increase in the number of accessible photocatalytic sites. However, Henry *et al.*^66^ subsequently demonstrated that increasing TiO_2_ thickness over a substantially wider range did not necessarily produce a corresponding increase in the paraffin-removal rate. This suggests that once sufficient photocatalytic surface area is available, additional catalyst thickness may provide diminishing returns.

Several factors may account for this behavior. Photocatalytic reactions are generally limited to regions that are effectively illuminated and accessible to the reactant. Increasing film thickness may therefore introduce catalyst material that contributes relatively little to the observed surface reaction. In addition, thicker films may exhibit different morphology, porosity, roughness, or mass-transport characteristics. The optical properties of both the photocatalyst and pollutant layer must also be considered.

These observations indicate that QCM studies should report photocatalyst loading and film thickness explicitly and, where possible, investigate more than one loading. Such experiments would allow the distinction between a catalyst-limited regime, in which additional photocatalyst increases the reaction rate, and a reaction- or transport-limited regime, in which further increases in catalyst loading provide little benefit.

### 5.3 Influence of pollutant loading and optical screening

The amount of pollutant deposited on the photocatalyst-coated QCM can substantially influence the measured response. At low pollutant loading, the deposited molecules may be readily accessible to reactive species and incident radiation. With increasing loading, however, the pollutant layer itself may become a barrier to light penetration and mass transport.

The soot experiments reported by Chin *et al.*^34^ provide a particularly clear example. At relatively low soot loading, almost complete oxidation was observed, whereas progressively higher soot loadings resulted in markedly slower oxidation. At sufficiently high loading, the soot layer strongly attenuated the incident UV radiation, limiting the effective irradiation of the photocatalyst. This result demonstrates that apparent photocatalytic efficiency can decrease with increasing pollutant loading even when the photocatalyst itself remains unchanged.

Consequently, comparisons between different QCM photocatalytic studies should take the pollutant loading into account. Reporting only the percentage degradation may be misleading when the initial surface loading, film thickness, or optical transmission differs substantially between experiments. QCM has an advantage in this respect because it permits the deposited mass to be determined directly, provided that the frequency response can be reliably converted into mass.

### 5.4 Influence of irradiation and reaction environment

Light wavelength and irradiance are fundamental parameters governing photocatalytic activity. Moreover, QCM measurements introduce additional environmental variables that may influence the measured frequency independently of chemical degradation.

The studies reviewed here demonstrate the importance of controlling temperature, relative humidity, oxygen concentration, pressure, and irradiation history. Henry *et al.*^66^ showed that the rate of paraffin removal depended on irradiation intensity and that oxygen availability influenced the photocatalytic reaction under selected conditions. Chin *et al.*^34^ demonstrated that UV transmission through the pollutant layer can become an important limitation at high soot loading. These observations indicate that irradiation conditions should be reported quantitatively instead of being simply described as “UV irradiation”.

Relative humidity is particularly important for QCM measurements conducted under ambient conditions. Manuda *et al.* demonstrated that TiO_2_-coated QCMs exhibited mass changes associated with atmospheric water under different relative-humidity conditions.^8^ Consequently, changes in humidity during a photocatalytic experiment can generate frequency shifts that may be incorrectly attributed to pollutant degradation. The use of a photocatalyst-only control and experiments at controlled humidity therefore provides an important means of separating water-related effects from pollutant degradation.

The influence of irradiation itself must also be considered. Illumination can alter the temperature of the QCM crystal and surrounding gas, potentially producing a frequency shift unrelated to photocatalysis. Reference-QCM configurations provide an effective approach for identifying and compensating for such background responses. Abe *et al.* demonstrated the value of a reference-QCM arrangement for correcting environmental frequency changes during irradiation,^32^ while Henry *et al.* employed reference measurements and control experiments to distinguish genuine photocatalytic responses from instrumental and environmental contributions.^50^

These observations suggest that a reliable QCM photocatalytic experiment should include a stable dark baseline, controlled irradiation conditions, and an appropriate reference or photocatalyst-only control wherever possible.

### 5.5 Reliability and limitations of QCM-based photocatalytic measurements

Although QCM provides a straightforward and highly sensitive means of measuring interfacial mass changes, the technique does not directly identify the chemical species responsible for a frequency shift. This represents the most important limitation when QCM is used as a standalone technique for photocatalytic analysis.

Under ideal conditions, a positive frequency shift during irradiation can be interpreted as a decrease in mass associated with the removal of adsorbed material. In practice, however, several simultaneous processes may contribute to the measured response, including pollutant degradation, adsorption and desorption of water, adsorption or desorption of reactants and intermediates, changes in film hydration, temperature-induced frequency shifts, and changes in the viscoelastic properties of the coating.

The studies of Hidaka *et al.* illustrate the complexity of interpreting QCM responses in liquid-phase photocatalysis.^20,22^ Photoinduced adsorption and desorption can occur simultaneously with degradation, making it difficult to assign the complete frequency response to the disappearance of the original pollutant. Similarly, Manuda *et al.* demonstrated that surface-water removal can contribute substantially to the apparent mass loss under atmospheric conditions.^8^

Another limitation arises from the Sauerbrey relationship itself. The direct conversion of frequency change into mass assumes that the deposited layer behaves approximately as a rigid, laterally homogeneous film that is sufficiently coupled to the oscillating crystal. These assumptions may become invalid for soft, hydrated, porous, viscoelastic, or partially detached photocatalytic films. Under such circumstances, a frequency shift may reflect not only mass change but also changes in the mechanical properties of the interfacial layer.

QCM-D can help address this limitation by simultaneously measuring changes in resonance frequency and energy dissipation. Changes in dissipation provide additional information about the viscoelastic and hydration state of the interfacial layer and may therefore help distinguish rigid mass loss from changes in film structure. However, QCM-D does not by itself provide chemical identification, and appropriate viscoelastic modelling and complementary analytical techniques remain necessary.

For these reasons, the reliability of QCM-based photocatalytic measurements is strongly dependent on experimental controls. A photocatalyst-only control, pollutant-only control where appropriate, dark control, and reference QCM can provide valuable information about non-photocatalytic contributions. Independent measurements using spectroscopic or chromatographic methods are also desirable when the objective is to demonstrate chemical degradation instead of mass removal.^74^Table 3 summarizes and compares the key methodological traits and limitations identified across the studies described above.

**Table 3:** Critical comparison of methodological features and limitations of QCM-based photocatalytic studies

Reference	Reference/background control	Continuous monitoring during irradiation	Adsorption separated before irradiation	Environmental variables controlled	Independent validation	Major limitation/strength
35	Limited	No	Partly	UV controlled	SEM	Early demonstration; intermittent measurements
17	Bare/modified QCM comparison	No	Yes	Temperature concern	XPS	UV irradiation interrupted every 10 min
47	Bare QCM comparison	No/limited	Yes	UV controlled	Surface wettability	Mainly self-cleaning/wettability assessment
50	Reference QCM	Yes	Yes	Temperature, pressure	Control experiments	Strong quantitative methodology
34	Bare/TiO_2_ comparisons	Yes	Yes	RH, temperature, pressure, UV transmission	Mass balance + kinetic model	Strong optical-screening effect at high soot loading
32	Reference QCM array	Yes	Yes	UV/environmental compensation	Comparison with conventional QCM	High sensitivity makes environmental artefacts visible
52	Bare QCM comparison	Periodic	Yes	Atmosphere/vacuum	No	Interrupted irradiation; long experiment
8	TiO_2_-only background	Yes	Yes	RH explicitly studied	XPS + Raman	Surface-water contribution substantial
74	Background/control	Yes	Yes	UV/visible irradiations compared	UV-Vis spectroscopy	Intermediate formation complicates mass interpretation
21 and 48	Adsorption/rinsing steps	Yes	Yes	pH, temperature	No	Strong controlled aqueous methodology
22	Limited	Yes	No	pH controlled	Fluorimetry	Photoinduced adsorption/desorption confounds degradation
75	Bare-QCM comparison	Yes	Partly	pH, scavenger conditions	Separate adsorption controls	Mechanism of deposition changes not fully resolved
77	No	Yes	Yes	Water/O_2_ conditions	No	QCM-D resolves viscoelastic contributions

### 5.6 Reproducibility and methodological standardization

A further limitation of the current literature is the lack of consistent reporting of experimental parameters. The reviewed studies employ different QCM frequencies, electrode materials, coating procedures, photocatalyst thicknesses, pollutant loadings, irradiation wavelengths and intensities, environmental conditions, and data-analysis methods. In addition, the number of independent measurements and associated uncertainties are not consistently reported.

This lack of standardization limits quantitative comparison between research groups. For example, a frequency shift measured using a 5 MHz crystal cannot be directly compared with an equivalent frequency shift measured using a 10 or 17 MHz crystal without considering the corresponding mass sensitivity. Similarly, percentage mass removal cannot be meaningfully compared when the initial pollutant loading differs substantially.

Future QCM-photocatalytic studies should therefore report, at minimum, the fundamental resonance frequency, electrode material and active area, harmonic used, mass sensitivity or calibration procedure, photocatalyst composition and loading, film thickness, pollutant identity and initial loading, irradiation wavelength and irradiance, exposure time, temperature, relative humidity or solvent conditions, oxygen concentration where relevant, and the procedure used for converting frequency changes into mass. These recommendations are elaborated in Table 4.

**Table 4:** Recommended minimum reporting parameters for QCM-based photocatalytic studies

Category	Parameter recommended for reporting
QCM	Fundamental frequency
	Crystal/electrode material
	Active electrode area
	Mass sensitivity/calibration
	Harmonic used
Photocatalyst	Chemical composition
	Loading
	Film thickness
	Deposition method
Pollutant	Identity
	Initial concentration/loading
	Surface loading where applicable
Irradiation	Wavelength
	Irradiance
	Exposure time
Environment	Temperature
	Relative humidity/solvent
	O_2_ concentration/partial pressure
Measurement	Dark baseline
	Reference/background QCM
	Continuous/intermittent mode
Analysis	Sauerbrey/viscoelastic model
	Kinetic model
Reproducibility	Number of independent measurements
	Uncertainty/error
Validation	Independent chemical/spectroscopic method
Interpretation	Mass loss *vs.* chemical degradation explicitly distinguished

The use of replicate measurements and explicit uncertainty estimates should also become routine. Where possible, QCM results should be supported by an independent method capable of confirming the chemical transformation of the pollutant. Such standardization would greatly improve the reproducibility and comparability of QCM-based photocatalytic investigations.

### 5.7 Emerging photocatalytic systems as opportunities for QCM investigation

The literature identified in this review remains concentrated largely on conventional semiconductor photocatalysts, particularly TiO_2_ and related oxide systems. Several rapidly developing photocatalytic material classes therefore represent important opportunities for extending QCM-based investigations.

Metal–organic frameworks (MOFs) and MXene-based materials have already been investigated using QCM in adsorption and sensing applications, demonstrating that these materials can be integrated with acoustic sensing platforms.^78,79^ However, these applications are distinct from using QCM to directly monitor photocatalytic degradation. The high surface area and strong adsorption properties of MOFs make them particularly interesting candidates for QCM studies because adsorption and photocatalytic transformation could potentially be monitored within the same experiment. Similarly, the surface chemistry and two-dimensional morphology of MXenes may provide opportunities for studying pollutant adsorption and interfacial photocatalytic reactions using QCM.

Emerging single-atom photocatalysts represent another potentially important research direction. Their atomically dispersed active sites and distinctive surface reaction mechanisms could make QCM useful for investigating changes in interfacial pollutant loading during photocatalytic reactions. Nevertheless, no convincing study specifically applying QCM to monitor photocatalytic degradation by a single-atom photocatalyst was identified in the literature search conducted for this review. This should therefore be regarded as a research opportunity.

A similar distinction is required for plasmonic photocatalysts. QCM has been combined with plasmonic and localized surface plasmon resonance measurements for interfacial sensing and adsorption studies.^80^ However, these applications should not be considered equivalent to QCM monitoring of plasmon-enhanced photocatalytic degradation. The combination of acoustic and optical measurements could nevertheless be particularly valuable for plasmonic photocatalysis as optical excitation, photothermal effects, adsorption, and chemical transformation may occur simultaneously.

Z-scheme and S-scheme systems should be considered as charge-transfer architectures instead of independent material classes. A recent study incorporated QCM into the characterization of a semiartificial colloidal Z-scheme system, although QCM was used to investigate interfacial enzyme adsorption instead of directly monitoring photocatalytic degradation.^81^ No convincing example was identified in which QCM was used specifically to quantify photocatalytic degradation of a system explicitly described by the authors as an S-scheme photocatalyst. QCM therefore has potential to provide complementary interfacial information for these systems, while it cannot independently establish the charge-transfer mechanism. Combining QCM with techniques such as *in situ* XPS, Kelvin probe measurements, transient absorption spectroscopy, electron paramagnetic resonance, or surface photovoltage measurements would be necessary for a more complete mechanistic interpretation.

Overall, these emerging material systems demonstrate that the future value of QCM is unlikely to lie simply in applying the technique to an increasing number of photocatalysts. Instead, its greatest contribution may arise from combining interfacial mass measurements with complementary measurements capable of identifying chemical transformation, charge–transfer processes, and changes in interfacial structure.

## 6. Conclusions and future Prospects

QCM provides a versatile and highly sensitive approach for monitoring interfacial mass changes associated with photocatalytic processes. The studies reviewed in this work demonstrate that QCM can be applied at both solid-air and solid–liquid interfaces and can provide real-time information on pollutant adsorption and removal under irradiation. In particular, the technique offers an important advantage over conventional bulk analytical methods by directly monitoring changes occurring at the photocatalyst-coated surface.

Nevertheless, the literature also demonstrates that a QCM frequency response should not automatically be equated with photocatalytic degradation. Adsorption, desorption, water uptake and removal, temperature changes, changes in film viscoelasticity, and other environmental effects may contribute to the observed response. The reliability of QCM-based photocatalytic measurements therefore depends strongly on appropriate controls, stable experimental conditions, reference measurements, and complementary analytical techniques. The absence of standardized reporting practices currently limits quantitative comparison between studies.

Future developments should therefore focus on improving both the sensitivity and the interpretability of QCM measurements. Reference-QCM configurations provide an effective approach for compensating for frequency shifts caused by irradiation and environmental fluctuations.^82,83^ Higher-frequency acoustic resonators can further improve mass sensitivity, potentially enabling the detection of smaller interfacial mass changes.^84^ QCM-D offers an additional dimension by simultaneously measuring frequency and dissipation responses, allowing changes in interfacial mass to be evaluated together with changes in film viscoelasticity and hydration.^85^ Appropriate viscoelastic modelling may therefore be particularly valuable for photocatalytic coatings that are porous, hydrated, or mechanically soft.^86^

Integration with microfluidic platforms represents another promising direction. Microfluidic QCM systems could provide precise control over pollutant concentration, flow rate, residence time, dissolved oxygen, pH, and other reaction parameters while reducing sample volume.^87^ Such configurations could improve reproducibility and facilitate controlled *operando* investigations of photocatalytic reactions under well-defined conditions.

Beyond conventional QCM, film bulk acoustic resonators (FBARs) and nanoelectromechanical systems (NEMS) provide opportunities for operation at substantially higher frequencies and enhanced mass sensitivity.^88,89^ Although these technologies have been extensively developed for sensing applications, their application to photocatalytic systems remains comparatively underexplored. Their integration with photocatalytic coatings could provide access to smaller and faster interfacial mass changes, provided that challenges associated with optical excitation, thermal effects, coating stability, and calibration are appropriately addressed.

Multimodal characterization is likely to be particularly important for the future development of QCM-based photocatalytic analysis.^90^ QCM or QCM-D could be combined with Raman spectroscopy, infrared spectroscopy, UV-visible spectroscopy, X-ray photoelectron spectroscopy, X-ray absorption spectroscopy, or electron paramagnetic resonance to simultaneously monitor interfacial mass changes and chemical or electronic transformations. Such combinations are important because QCM alone cannot establish the chemical identity of the species responsible for a measured mass change.^91^ As an example, a decrease in mass may arise from pollutant degradation, water removal, desorption of an intermediate, or restructuring of the photocatalytic film. Independent chemical information can therefore significantly strengthen the interpretation of QCM data.

*Operando* QCM measurements combined with spectroscopy may also provide a route toward distinguishing adsorption, surface reaction, intermediate formation, and final product removal during photocatalysis.^92^ This would represent a major advance over the conventional interpretation of a monotonic frequency shift as evidence of degradation. In particular, combining QCM-D with spectroscopic measurements could allow simultaneous investigation of mass, mechanical, optical, and chemical changes at the photocatalyst interface.

Data analysis is another area in which substantial progress can be expected. QCM photocatalytic experiments generate time-dependent responses that may contain multiple overlapping processes, including adsorption, desorption, reaction, environmental fluctuations, and changes in film properties. Artificial intelligence and machine-learning (AI/ML) approaches could potentially assist in separating these contributions when sufficiently large and well-characterized datasets become available.^93^ However, such approaches should be developed in parallel to physically meaningful models.

The expansion of QCM-based photocatalytic research toward emerging materials also represents an important opportunity. MOFs, MXenes, single-atom photocatalysts, and plasmonic systems have demonstrated distinctive adsorption, electronic, or catalytic properties yet remain largely unexplored from the perspective of direct QCM monitoring of photocatalytic degradation. Similarly, QCM could provide complementary interfacial information for emerging Z-scheme and S-scheme heterojunction architectures when combined with techniques capable of resolving charge–transfer processes. These applications could be particularly valuable for distinguishing enhanced adsorption from genuine enhancement of surface reaction kinetics.

Ultimately, the future role of QCM in photocatalysis should extend beyond the measurement of frequency shifts. The combination of higher-frequency resonators, reference compensation, QCM-D, microfluidics, FBAR/NEMS technologies, multimodal spectroscopy, and advanced data analysis could transform QCM from a sensitive mass-monitoring technique into a quantitative *operando* platform for investigating adsorption, interfacial reaction, and structural transformation during photocatalysis. Establishing standardized experimental and reporting protocols will be essential for achieving this objective and for enabling meaningful comparison of photocatalytic performance across different materials and research groups.

In conclusion, QCM occupies a distinctive position among photocatalytic characterization techniques as it directly probes interfacial mass changes with high temporal sensitivity. Its limitations, particularly the inability to identify chemical species from frequency changes alone, should be recognized rather than overlooked. When appropriately controlled and combined with complementary analytical methods, QCM has considerable potential to provide mechanistic insight into photocatalytic processes and to address interfacial phenomena that are difficult to resolve using conventional bulk analytical techniques.

## Author contributions

K. R. Jaliya Manuda: conceptualization, writing – original draft. Dilushan R. Jayasundara: conceptualization, writing – review and editing, supervision.

## Conflicts of interest

The authors declare no conflicts of interest.

## Data Availability

No primary research results, software or code have been included and no new data were generated or analysed as part of this review.
